# Anaerobic Fungal Mevalonate Pathway Genomic Biases Lead to Heterologous Toxicity Underpredicted by Codon Adaptation Indices

**DOI:** 10.3390/microorganisms9091986

**Published:** 2021-09-18

**Authors:** Ethan T. Hillman, Elizabeth M. Frazier, Evan K. Shank, Adrian N. Ortiz-Velez, Jacob A. Englaender, Kevin V. Solomon

**Affiliations:** 1 Department of Agricultural and Biological Engineering, Purdue University, West Lafayette, IN 47906, USA; ehillman@umich.edu (E.T.H.); frazie34@purdue.edu (E.M.F.); Aortizvelez7438@sdsu.edu (A.N.O.-V.); jaenglaender@gmail.com (J.A.E.); 2 Purdue University Interdisciplinary Life Sciences, Purdue University, West Lafayette, IN 47906, USA; 3 Department of Biological Sciences, Purdue University, West Lafayette, IN 47906, USA; shank3@purdue.edu

**Keywords:** anaerobic fungi, mevalonate pathway, isoprenoid biosynthesis, heterologous expression, codon usage, codon optimization

## Abstract

Anaerobic fungi are emerging biotechnology platforms with genomes rich in biosynthetic potential. Yet, the heterologous expression of their biosynthetic pathways has had limited success in model hosts like *E. coli*. We find one reason for this is that the genome composition of anaerobic fungi like *P. indianae* are extremely AT-biased with a particular preference for rare and semi-rare AT-rich tRNAs in *E coli*, which are not explicitly predicted by standard codon adaptation indices (CAI). Native *P. indianae* genes with these extreme biases create drastic growth defects in *E. coli* (up to 69% reduction in growth), which is not seen in genes from other organisms with similar CAIs. However, codon optimization rescues growth, allowing for gene evaluation. In this manner, we demonstrate that anaerobic fungal homologs such as *PI.atoB* are more active than *S. cerevisiae* homologs in a hybrid pathway, increasing the production of mevalonate up to 2.5 g/L (more than two-fold) and reducing waste carbon to acetate by ~90% under the conditions tested. This work demonstrates the bioproduction potential of anaerobic fungal enzyme homologs and how the analysis of codon utilization enables the study of otherwise difficult to express genes that have applications in biocatalysis and natural product discovery.

## 1. Introduction

Over the last century, fungi have been a source of valuable products in biotechnology, from medicines and insecticides to food additives and enzymes [1]. Anaerobic fungi in particular are increasingly recognized as a vast, untapped source of enzymes for biotechnology [2,3,4]. Genomic studies of anaerobic fungi show they have the largest array of biomass-degrading enzymes among fungi, which can be used for platforms that convert lignocellulosic waste to biofuels and chemicals [3,5]. In addition to plant biomass-degrading enzymes, anaerobic fungi have specialized membrane transporters [6] and unique biosynthetic pathways [7,8] that may be a valuable source of natural products, like polyketides synthases (PKS), non-ribosomal peptide synthetases (NRPS), terpenes, and bacteriocins [1,9]. However, the full potential of anaerobic fungi remains unrealized because there are few existing tools for their engineering.

Heterologous expression partially addresses this gap by evaluating and using promising genes from hard-to-engineer organisms in more amenable model organisms such as *E. coli* and *S. cerevisiae* [10]. These strategies have been used to make products in the food, pharmaceutical, and agricultural industries [11]. While heterologous expression is a very powerful tool, the evaluation of many proteins have been limited by potential pitfalls, such as codon bias [12], post-translation modification [13], and dissimilarity of host environment [14,15], that can cause expression to fail [16]. Previous attempts to heterologously express anaerobic fungal genes have often encountered similar issues in both *E. coli* and *S. cerevisiae* [6,13,17]. However, *E. coli* has successfully been used as a host for production of several proteins from anaerobic fungi, including cyclophilins, cellulases, and both hydrogenosomal, and scaffoldin [2,9]. Despite these successes, issues related to the expression of multiple anaerobic fungal proteins or full biosynthetic pathways in *E. coli*. have not been resolved.

One of the factors that limits the heterologous expression of anaerobic fungal genes in these hosts is that the genome composition of anaerobic fungi is divergent from most model organisms. The genomes of anaerobic fungi have the lowest %GC in the fungal kingdom (~20% GC) and are largely AT-rich in both the intergenic and coding regions [18]. For example, in the recently isolated anaerobic fungus *Piromyces indianae,* or *Piromyces* sp. UH3-1 [19,20], the AT-rich regulatory regions of DNA skew the overall %GC lower, while the coding regions remain relatively AT-biased at around 27.9% GC. On the other hand, the genomes of the model organisms *E. coli* and *S. cerevisiae* are more balanced at around 50% and 38% GC, respectively, with little difference between coding and intergenic regions [21,22]. The disparities in gene composition between anaerobic fungi and the underlying codon utilization of model hosts have hindered heterologous expression of anaerobic fungal genes from traditionally cloned genes [13]. The overuse of low abundance codons in model organisms with dissimilar codon usage is anticipated to impact cell health by depleting tRNA pools vital to the expression of essential genes like those involved in cell proliferation [23]. Synthesis of codon-optimized genes enables us to efficiently overcome the barriers that hinder our ability to evaluate and tap the potential of anaerobic fungi with a variety of model yeast and bacterial hosts, including *E. coli*.

In this study, we evaluated the effects of expressing native and codon optimized genes from anaerobic fungi in *E. coli*. We focused on expressing individual genes and the complete mevalonate biosynthesis pathway from recently isolated *Piromyces indianae,* a pathway composed of three genes (*atoB*, *HMGS*, and *HMGR*) that convert acetyl-CoA into mevalonate, a valuable terpenoid precursor. We demonstrate that, without codon optimization, some genes from anaerobic fungi cause moderate to severe growth defects in *E. coli* that are not predicted by the standard codon adaptation index. Additionally, these defects are evident even in strains designed to express difficult genes composed of rare *E. coli* codons. Further, we provide evidence that codon optimization enables *E. coli* to produce compounds from the biosynthesis pathways of anaerobic fungi, and that specific *P. indianae* homologs increased production approximately 2.5-fold compared to the heterologous pathway with canonical yeast homologs from *S. cerevisiae*. While further optimization of these homologs may be achieved by exchanging promoters of different strength, the work demonstrated here provides a rationale for revisiting anaerobic fungal enzymes that previously failed to heterologously express in order to fully evaluate their biotechnological capacity for biofuels and bioproduction.

## 2. Materials and Methods

### 2.1. Homolog Identification, Primer Design, PCR, RT-PCR, and Cloning

Using the Search terms “acetoacetyl-CoA transferase”, “hydroxymethylglutryl-CoA synthetase”, and “hydroxymethylglutryl-CoA reductase,”the genomic databse of Neocallimastgomycota on Mycocosm [24] was searched. The coding regions of the resulting protein sequences were analyzed with BLAST [25] to confirm homologs. The coding sequence of the homologs were obtained from Mycocosm and aligned using MEGA7 [26]. Degenerate primers were designed to amplify the gene sequences from *Piromyces indianae* (*Piromyces* sp. *UH3-1*) [19]. PCR amplicons were sequenced to confirm target identity of the *atoB*, *HMGS*, or *HMGR* homolog. To isolate genes without introns for cloning, genes were amplified from a cDNA library. Total RNA was extracted from *Piromyces indianae* using the QIAGEN (Germantown, MD, USA) AllPrep Fungal DNA/RNA/Protein kit. Reverse transcription PCR was performed using the QIAGEN QuantiTect Reverse Transcription Kit along with random octamers according to the manufacturer’s instructions. From the resulting cDNA pool, the target genes were PCR amplified with primers flanked by a 5′ BglII or XmaI site and a 3′ XhoI, BcuI, or NotI site (Table S4). Finally, these amplicons were digested with the flanking enzymes and ligated with Rapid T4 ligase (ThermoFisher; Waltham, MA, USA) into the pETM6 backbone [27] digested with the same enzymes and dephosphorylated with calf intestinal alkaline phosphatase (CIAP). Plasmids were transformed by heat shock into chemically competent DH5 *E. coli* cells (Table S5) and plasmids were subsequently isolated and sequenced to confirm the constructs were correct. Following the ePathBrick [27] design, the full pathway was constructed on the backbone by first joining the *atoB* and *HMGS* homolog in one round of cloning, and joining the *HMGR* homolog with the first two genes in a final step. The analogous yeast pathway [28] was obtained from Addgene as a benchmark. In a manner similar to the original construction of the pathway, combinations of the yeast and *Piromyces* homologs were made to assess the individual productivities of the various homologs. Similarly, vectors with weaker or stronger T7 promoter derivates were generated and combined in an attempt to enhance mevalonate production. Because a yeast homolog vector organized with the *atoB*_H9__*HMGS*_C4__*HMGR*_H9_ pathway was found to be a high-producing stain, the *Piromyces* homolog pathways were constructed to match this promoter structure. All gene sequences and plasmids used in this study are listed in Tables S6 and S7, respectively.

### 2.2. Growth Analysis

Vectors containing the homologs for the *PI.atoB*, *PI.HMGS*, or *PI.HMGR* gene were each transformed into an electrocompetent BL21 (DE3) *E. coli* strain for expression from the T7 promoter (Invitrogen; Waltham, MA, USA). Full and intermediate pathway vectors were transformed similarly. Overnight starter cultures were inoculated from single colonies and used to inoculate fresh LB/ampicillin 5 mL cultures to OD 0.05 the following day. The initial O.D. was adjusted to approximately 0.05 and IPTG was added to 100 µM at the time of inoculation. Cultures were grown at 37 °C while shaking at 250 rpm in parallel with uninduced cultures for approximately three hours with the O.D. being measured every 30 min on a NanoPhotometer^®^ NP80 (Implen; Westlake Village, CA, USA). Percent relative growth was calculated by taking the final OD_600_ of the induced gene and dividing it by the final OD_600_ of the uninduced gene across the three-hour experiment. *E. coli* BL21 CodonPlus (DE3) RIPL strains (Invitrogen, Waltham, MA), which carry additional tRNA for arginine, isoleucine, proline, and leucine, were also transformed and grown in the same manner as described above, with the addition of chloramphenicol (50 μg/mL) in accordance with the CmR marker on the RIPL vector. All growth experiments were carried out in triplicate with the mean and standard deviation plotted.

### 2.3. Codon Adaptation Index, Codon Usage, and Codon Optimization

The protein encoding sequences were downloaded for *E. coli* (GenBank Accession #: U00096.3) and *P. indianae* genomes (Mycocosm: piromy1, https://genome.jgi.doe.gov/portal/Piromy1/, accessed on 14 March 2021). Using a python package developed by Lee et al. (2018), the CAI of the *E. coli* genome and *P. indianae* genomes were calculated from the coding sequences. The CAI histograms and the Gaussian distribution fit were calculated with PRISM v9.0. To calculate the codon usages, a BioPython [29] script was used to split the coding sequences every three base pairs to create trinucleotide codons for every gene. The codon occurrences were tallied and frequencies were calculated with respect to all trinucleotides (“overall usage”) and those that code for the same amino acid (“relative usage”). Trinucleotide frequencies, or codon usages, were compared for relative and overall usage between *E. coli* and *P. indianae*. Additionally, the codon usage of *E. coli* was used to determine the codon frequencies that should be used for optimizing *P. indianae* genes. The python script written for this analysis was uploaded to https://github.com/ehillman26/CodonAnalysisForAnaerobicFungi. These gene sequences were manually optimized by removing any rare codons and replacing both rare and infrequently used codons with frequently used codons so that the overall codon frequency of each gene roughly matched the *E. coli* relative codon usage. Genes were then synthesized by Twist Biosciences and cloned into the pETM6 vector as described above (see Section 2.1). CAI distributions and codon usage data were analyzed and plotted with GraphPad Prism v9.0.0.

### 2.4. Mevalonate Production Cultures and HPLC Analysis

Overnight cultures of BL21 (DE3) strains containing variants of the yeast, *Piromyces,* or hybrid mevalonate pathways were grown in LB/amp at 37 °C. For mevalonate production, cultures were inoculated to an O.D. of 0.05 in 2YT media (16 g/L tryptone, 10 g/L yeast extract, 5 g/L NaCl, 1% glycerol; pH~7.5). Then, small-scale (5 mL) 2YT cultures were grown at 37 °C while shaking at 250 rpm. When cultures reached an O.D. of 1, they were induced to a final concentration of 500 µM IPTG and left overnight (Total~18 h). Final O.D. was measured, and 450 µL of cleared supernatant was mixed with 50 µL of 14% H_2_SO_4_ in order to convert produced mevalonate to mevalolactone for HPLC analysis [30]. The acidified supernatant was immediately chilled at 4 °C for a minimum of 1 h and analyzed by HPLC. Briefly, analysis of a 20 µL sample of the resulting supernatant was injected on an Agilent 1260 instrument with separation via a BioRad Aminex HPX-87H at 50 °C in 5 mM H_2_SO_4_ mobile phase (rate 0.600 mL/min). Detection was performed with a refractive index detector (RID) in (+) signal polarity mode at 45 °C. To evaluate mevalonate production at larger scales with better aeration, larger 2YT cultures (50 mL) were also grown under the same parameters as above but in baffled flasks. All mevalonate titer experiments were carried out in triplicate with the mean and standard deviation plotted.

## 3. Results and Discussion

### 3.1. Codon Usage and Preferences of P. indianae Are Strongly AT-Biased

**Figure 1:**
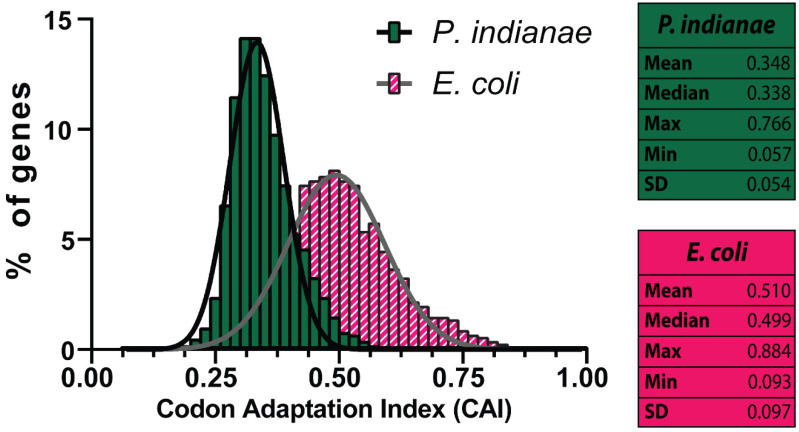
Distribution of Codon adaptation index (CAI) scores for *P. indianae* and *E. coli* genes with respect to their fitness in *E. coli*. Mean, median, maximum, and minimum scores are reported for the distributions. Normal Gaussian distributions are shown for each. SD = standard deviation.

The codon adaptation index (CAI) metric evaluates the potential expression level of a target gene based on its codon usage compared to that of highly expressed genes in the host of interest [31,32]. The CAI of every *P. indianae* gene using *E. coli* as the host was evaluated with a publicly available expression dataset [32], and the majority of *P. indianae* genes (>98%) were found to have *E. coli* CAI scores under 0.50 (Figure 1). The mean and median CAI of *P. indianae* genes are close to 0.35 and 0.34 (Figure 1), respectively, which is about 30% lower than the mean and median CAI of the *E. coli* genome (~0.50). In *E. coli*, highly expressed genes have a CAI of ~0.7 reflecting an optimal distribution of rare and abundant codons that does not overly deplete any individual tRNA pool. Moreover, rare codons have a functional role in protein elongation rates and folding. Thus, the exclusive use of abundant codons may be deleterious [33]. Given the low *E. coil* CAI of *P. indianae* genes, these genes are not likely to be highly expressed in *E. coli* [34]. It is not surprising to see that the mean and median of the *E. coli* genome are close to 0.50 given that its genome and codon usage are largely balanced (~50% GC) [21] and the distribution is a normal Gaussian distribution as seen across most genomes [35]. The *P. indianae* distribution is also relatively normal but has a left-skewed mean with lower standard deviation, suggesting it is strongly biased relative to *E. coli*. Furthermore, there are only ten *P. indianae* genes with a CAI of 0.60 or higher with the maximum CAI around 0.77. However, with the exception of two genes (ribosomal protein S29 and thioredoxin 2), these genes remain unannotated, hypothetical proteins, with no evidence for their evolutionary origins or function as determined by BLAST. While we can see most genes use different codons (Figure 1), the specific codons that are over- or under-utilized cannot be determined by CAI.

**Figure 2:**
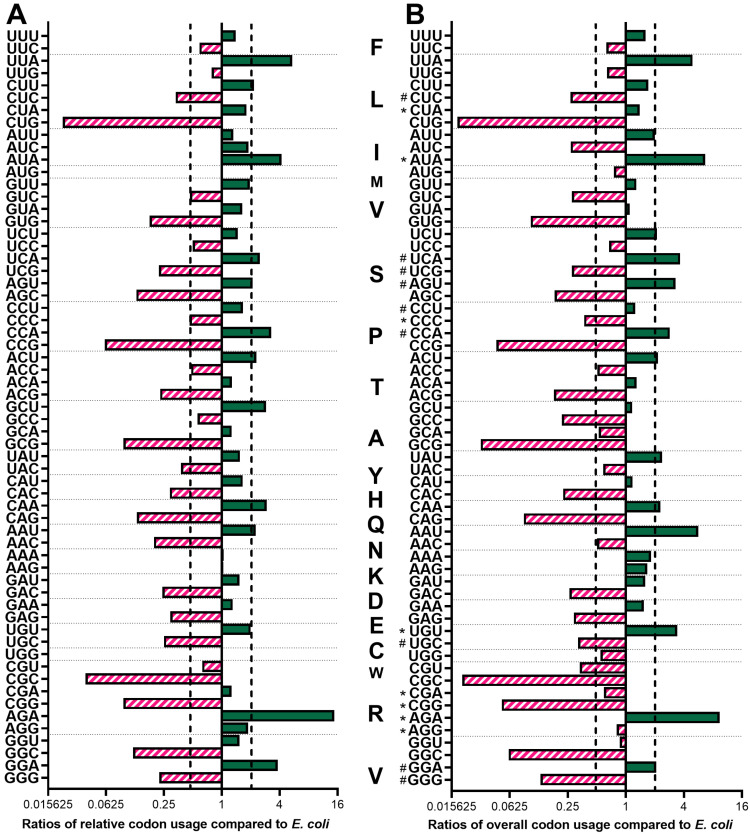
Relative and overall codon usage of *P. indianae* compared to *E. coli*. (**A**) Ratios of *P. indianae*’s codon usage relative to codons for the same amino acids compared to *E. coli*. (**B**) Ratios of *P. indianae*’s overall codon usage relative to all codons compared to *E. coli*. Ratios below 1 are shown in pink to indicate codons used more frequently in *E. coli* than *P. indianae*; ratios above 1 are shown in green to indicate codons used more frequently by *P. indianae* than *E. coli*. The vertical black dashed lines mark the 2-fold increase or decrease in codon use compared to *E. coli*. * and # indicate *E. coli* codons that are rare (≤0.5% overall usage) and semi-rare (≤1.0% overall usage), respectively. Delineations between codons encoding different amino acids are indicated by horizontal dashed gray lines.

To further explore what makes *P. indianae* genes poor fits for *E. coli*, we evaluated the relative codon usage for each amino and overall codon usage across the genome of *P. indianae*. The relative codon usage measures the preferred codon for an amino acid in an organism and is the frequency at which a particular codon is used relative to synonymous codons [33]. The overall usage, on the other hand, is the frequency at which a codon is used with respect to the codons for all amino acids. While *E. coli* uses a variety of AT-rich and GC-rich codons [21], anaerobic fungi are heavily biased towards AT-rich codons [18]. Our comparison of the relative usage of *E. coli* and *P. indianae* found that both species only preferred five codons in common out of the 18 amino acids with more than one codon (Figure 2); *S. cerevisiae* whose genome is somewhat AT-rich (~39% GC) and *E. coli*, in contrast, share eight of the same 18 preferred codons. The largest difference in preferred codons were for asparagine (Q), aspartate (N), arginine (R), proline (P), cysteine (C) and leucine (L) where codon usage of the *P. indianae* codon increased >40% compared to the frequency that *E. coli* uses this codon. In all cases, these biases reflect a preference for more AT-rich codons over GC alternatives favored by *E. coli*.

In addition to differences in synonymous codon preference, codon usage rates for preferred codons were markedly higher in *P. indianae*. Codon use was heavily biased towards use of the preferred codon in *P. indianae* 83 +/− 8% of the time. In contrast, *E. coli* and *S. cerevisiae* balance their usage of preferred codons at 57 +/− 9% and 58 +/− 8% of the time, respectively (Tables S1–S3). Unlike *E. coli* and other prokaryotes that compartmentalize codon usage using specific codons preferentially for highly expressed genes and other codons for specific processes such as cell division [23,31,35], anaerobic fungal codon usage is uniform across genes regardless of expression level, as is common among eukaryotes (data not shown). Thus, these biases suggest that anaerobic fungi more heavily prefer specific codons whose use is not correlated with expression level.

Anaerobic fungal codon biases are not limited to synonymous codon preferences. *P. indianae* genes overused 13 of the 61 amino-acid-calling codons compared to their overall usage in *E. coli* (>2-fold increase in the overall codon usage; Figure 2), which is similar to *S. cerevisiae* (14 overused codons compared to *E. coli*). Not surprisingly, these codons were all AT-rich. Out of these 13 codons, two are rare *E. coli* codons (AGA_R_ and AUA_I_; used in less than 0.5% of all codons) and two are semi-rare (UCA_S_ and AGU_S_; used in less than 1% of all codons). Frequent use of rare codons is problematic for expression in *E. coli* because it stalls translation and can lead to misfolded or truncated proteins [33]. Because these rare codons also have a special function in regulating the rate of protein synthesis and nascent chain folding [23,36], we suspect that over-expressed genes that consume the already small pools of rare codons could potentially tax the cells and slow down translation, and ultimately growth [37]. The most overused codons, AAU_N_, UUA_L_, and AAA_K_, however, are not rare codons. Collectively, they are overall used 18.9% of the time in *P. indianae* compared to 6.4% of the time in *E. coli* (Tables S1 and S2) and demonstrate there are also large differences in non-rare codon preferences. Work prior to this has demonstrated that tRNA pools of abundant codons increase with growth to help maximize cell health while the pools of non-abundant codons remain unchanged [38,39]. Much less work has been done to investigate how the overuse of non-abundant codons affects cell physiology [40]. However, it was previously shown that the overexpression of the *tnaC* gene from *E. coli* that contained semi-rare CCU_P_ codon lead to growth inhibition due to depleted CCU tRNA pools [41]. The genes of *P. indianae* are intriguing case studies because over a dozen of these codons are heavily used here. Overall, *P. indianae* genes use codons that are AT-rich and less frequent in *E. coli* and could potentially create translational burdens in heterologous hosts when overexpressed.

### 3.2. E.coli Are Not Well Equipped to Express the AT-rich Genes of P. indianae

**Figure 3:**
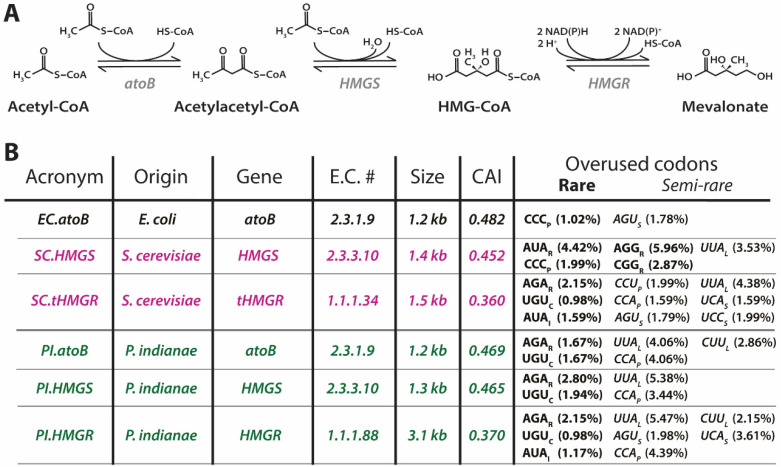
Mevalonate pathway and gene information. (**A**) Mevalonate biosynthesis pathway showing genes, cofactors, and substrate. (**B**) Homolog information for the genes evaluated in this study. Overused codons are used 2-fold or more in these genes compared to the host utilization. Rare and semi-rare codons are used 0.5% and 1.0% of the time or less, respectively. Overall codon usage of each gene is shown next to the codon. Gene names: acetyl-CoA acetyltransferase (*atoB*), HMG-CoA synthase (*HMGS*), and HMG-CoA reductase (*HMGR*).

**Figure 4:**
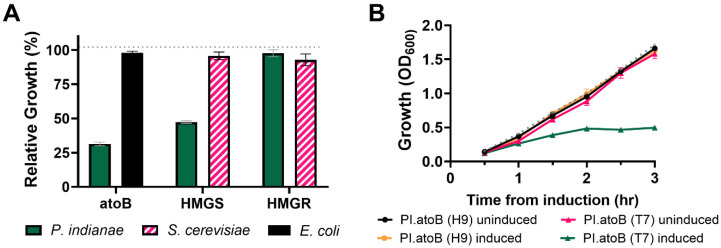
Relative growth of *E. coli* expressing the *E. coli*, *S. cerevisiae*, or *P. indianae* homologs. (**A**) Relative growth of *E. coli* BL21 when expressing individual genes of the mevalonate pathway under an inducible T7 promoter. (**B**) Growth curve of *E.coli* BL21 expressing the *PI.atoB* under the T7 promoter and a weaker H9 promoter variant. Growth is relativized to the growth of an uninduced control for each homolog. Relative growth is calculated by taking the final OD_600_ of the induced gene divided by the final OD_600_ of the uninduced gene. The T7-mCherry expression control is indicated by a dotted gray line in both panels A & B. Error bars represent standard deviation; n = 3 for all conditions.

To test the effects of expressing *P. indianae* genes in *E. coli*, we separately expressed three genes from a biosynthetic pathway from *P. indianae* (Figure 3A). We selected to study the mevalonate pathway (*atoB*, *HMGS*, and *HMGR*) as this is both a conserved pathway in fungi and because it can be used to support a broad range of isoprenoid bioproduction platforms [42]. Because this mevalonate pathway is not native to most bacteria including *E. coli*, characterization of these enzymes in this heterologous platform avoids substrate competition between the native pathways in model yeast hosts. Expression of these genes was compared to homologs from *S. cerevisiae* and *E. coli*, which have previously been expressed in *E. coli* [28]. Interestingly, the expression of *PI.atoB* and *PI.HMGS* reduced the growth of *E. coli* by about 69% and 53%, respectively (Figure 4), while expression of *PI.HMGR* had no effect on growth. These growth defects are unlikely to be the result of enzymatic activity because overexpressing the corresponding homologs from *E. coli* and *S. cerevisiae* (*EC.atoB, SC.HMGS, SC.HMGR*) does not result in similar growth reduction compared to the uninduced controls. The standard CAI metric also does not explain this discrepancy, as yeast homologs do not reduce growth despite having similar CAIs to those from *P. indianae* (Figure 3B).

Interestingly, we see that the *HMGR* homologs are nearly identical in both number of overused rare codons and their overall usage, and they both result in similar growth effects (Figure 4A). However, *PI.atoB* and *PI.HMGS* each only use two rare codons between 3–5% of the time, yet they have the largest effects on growth (Figure 4A) compared to *SC.HMGS*, *SC.HMGR*, and *PI.HMGR* that have three or more rare codons. Based solely on rare codons, it appears that fewer rare codons results in larger growth deficiencies for these genes. We suspect that the use of additional rare-codons may play a role in slowing down or stalling the translational machinery [33], which in turn slows the drain of the various overused tRNA pools, and thus limits growth defects. Additionally, codon content was recently shown to correlate with the mRNA levels where rare-codons could reduce protein expression by decreasing the stability of mRNA [40]. The AUA_I_ codon specifically was shown to have the largest effect at attenuating protein expression, and thus may play a role here in decreasing the lifespan of mRNA that overuse it, namely *SC.HMGS*, *SC.HMGR*, and *PI.HMGR*. In this case, even though these genes have the potential to overuse rare and semi-rare codons, the reduced mRNA levels keep the tRNA pools from being drained as quickly. In either case, it would seem that the overexpression of these proteins affects the physiology of the cell, which agrees with recent global proteomic analysis when proteins are overexpressed [43].

This translational burden and growth defect can be alleviated somewhat by reducing promoter strength from a stronger T7 promoter to a weaker H9 variant [27]. Only induction with a strong T7 promoter results in a growth defect (Figure 4B). However, it should be noted that, regardless of the promoter, expression of the native *P. indianae* genes was not detectable via SDS-PAGE (Figure S1), suggesting that both strong and weak promoters resulted in weak or no expression. Growth defects have been similarly observed when cellulases from anaerobic fungi were expressed in *S. cerevisiae* [13], which may be the result of similar codon deficiencies. No defect was seen from inducing the mCherry fluorescent protein with a T7-promoter (dotted gray line, Figure 4A,B). Our work suggests that these deficiencies may be overcome with contemporary codon optimization/harmonization strategies and thus previously examined anaerobic fungal enzymes with negligible expression may need to be reevaluated. Regardless, CAI alone is insufficient to predict heterologous growth defects of anaerobic fungal enzymes due to their severe biases for rare AT-rich codons that *E. coli* cannot accommodate.

### 3.3. Strains with Additional tRNAs for Rare Codons Do Not Effectively Relieve the Burden of Expressing P. indianae Genes

**Figure 5:**
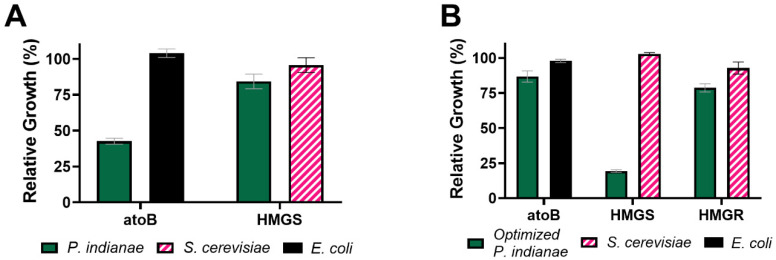
Relative growth in additional strains or with optimized genes. (**A**) Relative growth of *E.coli* BL21^+^ RIPL expressing the *E. coli*, *S. cerevisiae*, or *P. indianae* homologs of the mevalonate pathway. (**B**) Relative growth of *E. coli* BL21 expressing the *E. coli*-codon optimized versions of the *P. indianae* genes compared to the respective *atoB*, *HMGS*, or *HMGR* homolog. Relative growth is calculated by taking the final OD_600_ of the induced gene divided by the final OD_600_ of the uninduced gene. Error bars represent standard deviation; n = 3 for all conditions.

Strains of *E. coli* have been created to allow expression of proteins with codon mismatches by adding additional copies of rare tRNA genes. We evaluated if one of these strains, BL21-CodonPlus (DE3) RIPL (Agilent Technologies), that provides an additional tRNA copy for AGA_R_, AUA_I_, CCC_P_, and CUA_L_, could alleviate the growth defects of *P. indianae* homolog expression. Like other prokaryotes., the tRNA gene copy number and codon usage are highly correlated in the *E. coli* genome, ultimately allowing them to use codons with more abundant tRNAs at higher rates [33,44]. Therefore, increasing the available tRNA pools with additional copies of rare codons tRNA genes may allow increased expression of these genes where it was previously hindered. In this strain, the effect on *PI.HMGS* growth improved markedly, where growth was only reduced by about 16%. However, the *PI.atoB* homolog still reduced the growth about by 57% (Figure 5A). Looking at the specific codons supplemented by this strain, only AGA_R_ is highly used in these *P. indianae* homologs and makes up 1.7% and 2.8% of the overall codon usage for *atoB* and *HMGS*, respectively (Figure 3B). Because the other RIPL codons that are supplemented (AUA_I_, CCC_P_, and CUA_L_) do not alleviate the drain on the other overused codons, such as AAU_N_, AAA_K_, and UUA_L_, this strain is ill-equipped to efficiently express the *P. indianae* homologs and growth is still hindered.

### 3.4. Codon Optimization Alleviates the Growth Deficiencies Seen When the P. indianae atoB Is Expressed in E. coli

Because accommodating all of the codon deficiencies would require a large plasmid or an extensive strain engineering undertaking, we selected a codon harmonization approach to resolve the majority of the underlying codon issues. Codon harmonization matches the codon usage of each gene of interest to that of the *E. coli* genome to ensure proper protein folding and avoid potential stresses related to overusing any one codon–a strategy which is optimal for bacterial hosts [45]. When the codon optimized *P. indianae* homologs were expressed, we saw that the *PI.atoB* and *PI.HMGR* homologs had minor growth defects, growing to roughly 80% of the control level (Figure 5B). This suggests that by reducing the usage of suboptimal codons, *P. indianae* genes can be expressed without the same detrimental growth defects seen from the native unoptimized genes. Similarly, SDS-PAGE analysis demonstrates that the codon optimized genes express much better than the unoptimized genes (Figure S1). However, the growth defect of *PI.HMGS* observed after optimization was nearly twice as severe compared to the unoptimized gene. We suspected that at least part of the growth defect seen here is a result of increased protein activity when *PI.HMGS* in combination with the native *E. coli atoB* produce a toxic intermediate, hydroxy β-methylglutaryl-CoA (HMG-CoA), from acetyl-CoA pools [42,46]. The unoptimized *PI.HMGS*, however, does not produce this toxic effect possibly due to lower expression (Figure S1). As seen by another group expressing optimized genes from anaerobic fungi in *E.coli* [6], the growth deficiency may also result from improper folding or membrane incorporation. However, predictors of transmembrane domains [47] did not reveal the presence of any membrane-associated domains within *PI.HMGS* unlike the native *S. cerevisiae* homolog, which was previously truncated for expression in *E. coli* [28]. When the *PI.HMGS* gene was expressed as part of the complete pathway, we did not observe this same growth defect (see growth data, Figure S2) suggesting that the toxic HMG-CoA was being converted into mevalonate (see Section 3.6).

### 3.5. Expression of Unoptimized Genes Hinders Biosynthesis from P. indianae Genes

**Figure 6:**
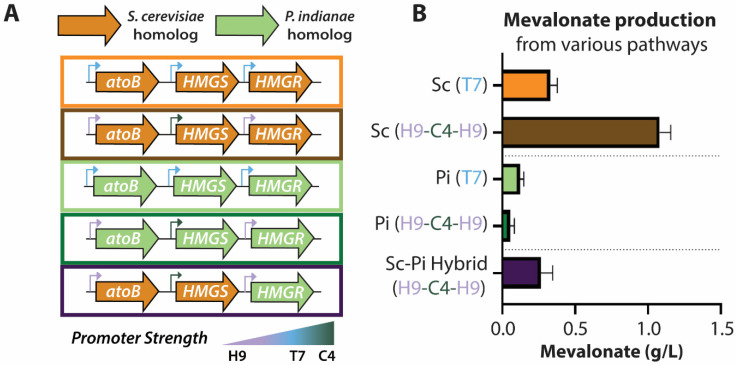
Mevalonate pathway variants and titers with unoptimized genes. (**A**) Different promoter and gene configurations of the Martin et al. (orange) and *P. indianae* (green) mevalonate pathways and a hybrid of them. (**B**) Mevalonate titers for the pathway variants after 20 h of culture; Sc = yeast, Pi = *P. indianae*; T7 = T7 promoters for each, H9 = H9-C4-H9 promoter configuration as shown in panel A. Error bars represent standard deviation; n = 3 for all conditions.

Despite the low CAI scores of the yeast homologs, expressing them in *E. coli* can produce mevalonate and subsequently terpenoids [28]. Initially, we evaluated the native mevalonate pathways (Figure 3A) of *S. cerevisiae*, *P. indianae*, and a hybrid of the two (Figure 6A) in order to understand if the native genes could produce mevalonate despite being poorly adapted for the *E. coli* chassis. To create the pathways, we took the individually cloned genes and combined them into one plasmid using the ePathBrick system [27]. In contrast to previous approaches that use an operonic approach to express all genes under one T7 promoter [28,48], we expressed each gene at independent levels via separate T7-inducible promoters of varying strength [49]. This also enabled the investigation of how changing the expression level of each enzyme with different promoter strengths affected the mevalonate output (see Figure 6A). We found that a common library variant with a *atoB*_H9_-*HMGS*_C4_-*HMGR*_H9,_ or H9-C4-H9, promoter architecture improved titers three-fold to ~1.0 g/L relative to initial reports with a T7 operon [28] (Figure 6B). The promoter strength of H9 is ~30% that of the consensus T7 promoter while C4 is a stronger promoter (~150% of consensus) [49]. In contrast to the yeast pathway, the native *P. indianae* pathway did not produce noteworthy amounts of mevalonate (<0.10 g/L) under either the all T7 or H9-C4-H9 promoter configuration (Figure 6A). While the low yields with the native *P. indianae* homologs are not surprising given the associated growth deficiencies of individual genes, the growth defects are only marginal when the genes are expressed together in the H9-C4-H9 configuration. The growth of the native pathway is similar to the pathway consisting of the codon optimized *P. indianae* homologs (Figure S2). As *PI.HMGR* expression was not associated with any growth defect in *E. coli*, we swapped this homolog for the *SC.HMGR* variant in the all yeast pathway and evaluated whether a hybrid of the yeast and *P. indianae* pathway (*EC.atoB*-*SC.HMGS*-*PI.HMGR*) could produce any mevalonate. Interestingly, we found that this hybrid pathway produced mevalonate titers similar to the yeast pathway under the T7 promoters (Figure 6B). Though the titers are not as high as the H9-C4-H9 yeast pathway that it was derived from, the production of even 0.25 g/L mevalonate is encouraging given that this gene was not codon optimized and came directly from *P. indianae*. Therefore, we evaluated pathways with codon optimized *P. indianae* homologs as a case study for how biosynthetic pathways from anaerobic fungi can be evaluated in *E. coli* for the discovery and production of valuable metabolites.

### 3.6. Codon Optimization Allows Heterologous Production of Mevalonate from P. indianae Enzymes

**Figure 7:**
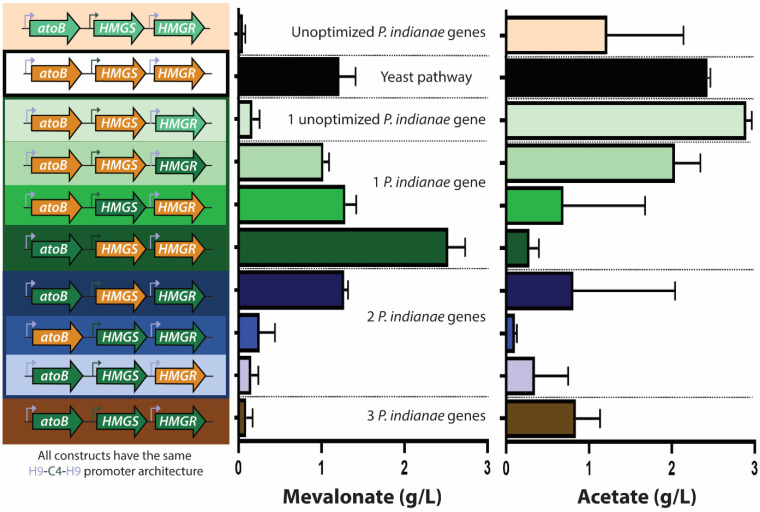
Mevalonate and acetate titers of original and hybrid mevalonate pathways. Pathway configuration (**left**), mevalonate production (**middle**), and acetate accumulation (**right**) from various mevalonate pathway hybrids containing individual genes from Martin et al. (orange), native *P. indianae* genes (light green), or *E. coli*-codon optimized *P. indianae* genes (dark green) after 20 h of culture. Green bars represent pathways with one PI homolog, blue bars and brown bars represent pathways with two and three PI homologs, respectively. All pathways are configured in the high-producing h9-promoter configuration. Errors bars represent standard deviation; n = 3 for all conditions.

Because codon optimization of the *P. indianae* genes relieved the growth deficiencies of the *atoB* gene, we evaluated if it also enabled increased production of mevalonate. Specifically, we investigated the production of the *P. indianae* pathway with both native and codon optimized genes, the yeast pathway, and hybrids thereof. In addition to mevalonate production, we also tracked the produced acetate to compare how these constructs affected the flux of the acetyl-CoA precursor to mevalonate or acetate. Hybrid pathways of yeast and *P. indianae* codon optimized genes were able to increase titers almost two-fold suggesting that codon optimized *P. indianae* homologs of the mevalonate pathway may be more catalytically active than yeast variants (Figure 7). In particular, *PI.atoB* improved mevalonate production by shunting acetyl-CoA flux away from acetate and toward mevalonate production, reducing acetate titers 88.4%. Both *PI.HMGR* and *PI.HMGS* in hybrid pathways resulted in similar titers of mevalonate when compared to the yeast pathway. However, both exhibited reduced acetate levels suggesting an accumulation of redirected carbon in the pathway. Pathways with native *P. indianae* genes (Figure 7, light green genes), on the other hand, produced reduced levels of mevalonate, similar to what we observed previously (<0.10 g/L). The native *PI.atoB* and *PI.HMGS* produced no mevalonate (data not shown) while the native *PI.HMGR* greatly reduced mevalonate production (~0.18 g/L) and led to higher accumulation of acetate (~3.0 g/L) compared to the yeast pathway (Figure 7, 1 unoptimized *P. indianae* gene). These results further demonstrate that genes from anaerobic fungi need to be codon optimized for both growth and mevalonate production in *E. coli*. Previously, individual genes from anaerobic fungi have been expressed and purified in *E. coli* after codon-optimization in order to be combined for a pretreatment cellulase cocktail for lignocellulose degradation [50]. However, this is the first demonstration of multiple genes being simultaneously expressed in the same host as is required for the mevalonate biosynthesis pathway.

Most hybrid constructs with two *P. indianae* genes (Figure 7, blue bars) saw a decrease in the amount of both acetate and mevalonate when compared to the parent one-*P. indianae*-gene hybrid (Figure 7, green bars). Mevalonate produced by combining the first and last gene homologs (*PI.atoB* and *PI.HMGR*) with the *SC.HMGS* reached a level between the levels produced by either one-gene hybrid. Based on the lower acetate level of this two-gene hybrid, we suspect that *SC.HMGS* was hindering the overall flux of acetyl-CoA to mevalonate compared to other two two-gene hybrids containing *PI.HMGS*. The *PI.atoB-PI.HMGS-SC.HMGR* pathway made very little mevalonate (less than 0.25 g/L), considering that these one-gene hybrids individually both produced more than 1 g/L. However, when we expressed this pathway on a smaller scale (5 mL, Figure S3), production titers increased to 1.6 g/L. While the reason for lower mevalonate titers at this larger scale (50 mL) is unclear, it suggests there may be specific culturing conditions that might improve the performance of these genes. Moreover, the optimal promoter architecture for anaerobic fungal homologs may be distinct from that of the yeast pathway tested here (H9-C4-H9) [51].

Ultimately, we show that codon optimization is necessary to unlock the biosynthetic potential of low-GC anaerobic fungi mirroring similar developments in high-GC *Streptomyces* and low-GC *Bacillus* for elucidation of bioactive natural products [52,53], anaerobic fungal membrane transporters [6], and even the use of reporters in *Staphylococcus* [54]. While many parameters remain to be tuned to obtain the highest possible mevalonate titers from the genes for anaerobic fungi, we demonstrate the functionality and utility of these *P. indianae* genes here. In spite of their low expression (see Section 3.2), which stymie classical catalytic characterization studies (i.e., k_cat_ and K*_m_* determination), the superior performance of hybrid *P. indianae* pathways suggests improved enzyme kinetics of anaerobic fungal homologs over the canonical yeast variants. Compared to the previous batch titers in *E.coli* using genes from *S. cerevisiae* (~0.4 g/L; [48]) or *Enterococcus faecalis* (3.1 g/L; [55]), our observed titers of 2.5 g/L are a promising starting point for future studies that focus on optimizing promoter organization and culture condition. Optimization strategies with existing genes, such as fed-batch fermentations and metabolic engineering, have yielded mevalonate yields up to 30 g/L [56]. These *P. indianae* sourced genes may prove to confer distinct advantages through anaerobic catalytic mechanisms that improve titers further [7]. In contrast to *SC.HMGR*, sequence homology analysis of *PI.HMGR* predicts that it uses NADH rather than NADPH as a cofactor in the synthesis of mevalonate (Figure 3A). While unvalidated, this putative cofactor preference may be beneficial for production in certain expression hosts and conditions.

## 4. Conclusions

In this study, we have validated the anaerobic fungal mevalonate pathway and revealed efficient homologs for heterologous expression in *E. coli* that synthesized mevalonate at levels comparable to or greater than known yeast homologs (up to 2.5 g/L). These active homologs also increased pathway efficiency by reducing carbon waste to acetate by almost 90% under the conditions tested. However, the intrinsic AT genomic bias of the source organism severely limits use of the native genes, which can inhibit heterologous host growth by up to 69% due to a preference for rare AT-rich codons. Sequence analysis is required to identify these biases as standard codon bias metrics, such as CAI, failed to predict the severe growth defects. Codon optimization, however, was an effective strategy to rescue growth and allow for gene evaluation of the *P. indianae* genes. Our work underscores the need for codon utilization analysis in harnessing genes from non-model organisms and lays the foundation for how additional biosynthetic pathways from anaerobic fungi can be expressed in model hosts like *E. coli*. Furthermore, codon optimization may be used as a strategy to revisit genes from anaerobic fungi that were previously abandoned after failed heterologous expression attempts. By overcoming these expression barriers, the repertoire of potentially valuable enzymes from anaerobic fungi can be fully realized.

## Data Availability

Not applicable.

## Supplementary Materials

The following are available online at https://www.mdpi.com/article/10.3390/microorganisms9091986/s1, Tables S1–S3—Codon Tables of *P. indianae*, *E. coli*, and *S. cerevisiae*, Figure S1—SDS-PAGE analysis of mevalonate homologs, Figure S2—Growth OD600 of 50 mL cultures containing various mevalonate pathways after 20 h of growth, Figure S3—Mevalonate and acetate titers of original and hybrid mevalonate pathways at 5 mL scale, Tables S4–S7—Strains, Oligos, Plasmids, and gene sequences used in this study.
