# Supplementary material for: Anaerobic Fungal Mevalonate Pathway Genomic Biases Lead to Heterologous Toxicity Underpredicted by Codon Adaptation Indices
Source: Microorganisms. 2021 Sep 18;9(9):1986. doi: 10.3390/microorganisms9091986 (PMC8468974; doi:10.3390/microorganisms9091986)
Supplement: Supplementary file 1 [file microorganisms-09-01986-s001.zip › microorganisms-1379661-supplementary.pdf]

SUPPLEMENTAL INFORMATION FOR

Genomic biases in the anaerobic fungal mevalonate pathway lead to heterologous toxicity underpredicted by codon adaptation indices

Ethan T. Hillman[1,2], Elizabeth Frazier [1], Evan Shank[3], Adrian Ortiz-Velez[1¥], Jacob Englaender[1†], and Kevin Solomon[1,2‡*]

[1] Department of Agricultural and Biological Engineering, Purdue University, West Lafayette, IN 47906, USA

[2] Purdue University Interdisciplinary Life Sciences, Purdue University, West Lafayette, IN 47906, USA

[3] Department of Biological Sciences, Purdue University, West Lafayette, IN 47906, USA

¥ Present Address: Department of Biology, San Diego State University, San Diego, CA 92182, USA

† Current Address: Agrospheres, INC., Charlottesville, VA 22901, USA

‡ Present address: Department of Chemical & Biomolecular Engineering, University of Delaware, Newark, DE 19716

* Correspondence: kvs@udel.edu

Table of contents:

- Tables S1–S3—Codon Tables of *P. indianae* , *E. coli,* and *S. cerevisiae*
- Figure S1—SDS-PAGE analysis of mevalonate homologs
- Figure S2 – Growth OD600 of 50 ml cultures containing various mevalonate pathways after 20 hours of growth
- Figure S3—Mevalonate and acetate titers of original and hybrid mevalonate pathways at 5ml scale
- Tables S4-7—Strains, oligos, plasmids, and gene sequences used in this study

Table S1 – Codon usage table for anaerobic fungus *Piromyces sp. UH3-1*. %Usage represents overall usage of the codon relative to the total of all codons used for all genes (coding sequences, CDS). Ratio = relative usage based on the frequency that a codon is used for the related amino acid.

	CODON	AMINO ACID	% USAGE	RATIO	CODON	AMINO ACID	% USAGE	RATIO	CODON	AMINO ACID	% USAGE	RATIO	CODON	AMINO ACID	% USAGE	RATIO	
**U**	UUU	Phe (F)	3.0%	0.71	UCU	Ser (S)	2.3%	0.28	UAU	Tyr (Y)	3.8%	0.82	UGU	Cys (C)	1.4%	0.86	**U**
	UUC	Phe (F)	1.1%	0.29	UCC	Ser (S)	0.7%	0.08	UAC	Tyr (Y)	0.8%	0.18	UGC	Cys (C)	0.2%	0.14	**C**
	UUA	Leu (L)	4.9%	0.60	UCA	Ser (S)	2.6%	0.30	UAA	STOP	0.1%	0.80	UGA	STOP	0.0%	0.09	**A**
	UUG	Leu (L)	0.7%	0.09	UCG	Ser (S)	0.2%	0.03	UAG	STOP	0.0%	0.11	UGG	Trp (W)	0.8%	1.00	**G**
**C**	CUU	Leu (L)	1.7%	0.22	CCU	Pro (P)	0.9%	0.27	CAU	His (H)	1.4%	0.86	CGU	Arg (R)	0.8%	0.26	**U**
	CUC	Leu (L)	0.2%	0.03	CCC	Pro (P)	0.1%	0.05	CAC	His (H)	0.2%	0.14	CGC	Arg (R)	0.0%	0.01	**C**
	CUA	Leu (L)	0.4%	0.05	CCA	Pro (P)	2.3%	0.65	CAA	Gln (Q)	3.0%	0.91	CGA	Arg (R)	0.2%	0.06	**A**
	CUG	Leu (L)	0.1%	0.01	CCG	Pro (P)	0.1%	0.03	CAG	Gln (Q)	0.3%	0.09	CGG	Arg (R)	0.0%	0.01	**G**
**A**	AUU	Ile (I)	5.4%	0.62	ACU	Thr (T)	2.6%	0.48	AAU	Asn (N)	9.0%	0.88	AGU	Ser (S)	2.3%	0.27	**U**
	AUC	Ile (I)	0.7%	0.09	ACC	Thr (T)	0.8%	0.15	AAC	Asn (N)	1.3%	0.12	AGC	Ser (S)	0.3%	0.03	**C**
	AUA	Ile (I)	2.7%	0.29	ACA	Thr (T)	1.8%	0.32	AAA	Lys (K)	7.0%	0.75	AGA	Arg (R)	1.9%	0.60	**A**
	AUG	Met (M)	2.0%	1.00	ACG	Thr (T)	0.2%	0.05	AAG	Lys (K)	2.0%	0.25	AGG	Arg (R)	0.2%	0.06	**G**
**G**	GUU	Val (V)	2.6%	0.57	GCU	Ala (A)	2.1%	0.55	GAU	Asp (D)	5.3%	0.90	GGU	Gly (G)	2.4%	0.58	**U**
	GUC	Val (V)	0.4%	0.09	GCC	Ala (A)	0.5%	0.14	GAC	Asp (D)	0.6%	0.10	GGC	Gly (G)	0.2%	0.05	**C**
	GUA	Val (V)	1.3%	0.28	GCA	Ala (A)	1.1%	0.28	GAA	Glu (E)	6.7%	0.91	GGA	Gly (G)	1.4%	0.34	**A**
	GUG	Val (V)	0.2%	0.06	GCG	Ala (A)	0.1%	0.03	GAG	Glu (E)	0.5%	0.09	GGG	Gly (G)	0.1%	0.03	**G**
	**U**				**C**				**A**				**G**				

Table S2 – Codon usage table for *E. coli*. %Usage represents overall usage of the codon relative to the total of all codons used for all genes (coding sequences, CDS). Ratio = relative usage based on the frequency that a codon is used for the related amino acid.

	CODON	AMINO ACID	% USAGE	RATIO	CODON	AMINO ACID	% USAGE	RATIO	CODON	AMINO ACID	% USAGE	RATIO	CODON	AMINO ACID	% USAGE	RATIO	
U	UUU	Phe (F)	1.9%	0.51	UCU	Ser (S)	1.1%	0.19	UAU	Tyr (Y)	1.6%	0.53	UGU	Cys (C)	0.4%	0.43	U
	UUC	Phe (F)	1.8%	0.49	UCC	Ser (S)	1.0%	0.17	UAC	Tyr (Y)	1.4%	0.47	UGC	Cys (C)	0.6%	0.57	C
	UUA	Leu (L)	1.0%	0.11	UCA	Ser (S)	0.7%	0.12	UAA	STOP	0.2%	0.62	UGA	STOP	0.1%	0.30	A
	UUG	Leu (L)	1.1%	0.11	UCG	Ser (S)	0.8%	0.13	UAG	STOP	0.03%	0.09	UGG	Trp (W)	1.4%	1.00	G
C	CUU	Leu (L)	1.0%	0.10	CCU	Pro (P)	0.7%	0.16	CAU	His (H)	1.2%	0.52	CGU	Arg (R)	2.4%	0.42	U
	CUC	Leu (L)	0.9%	0.10	CCC	Pro (P)	0.4%	0.10	CAC	His (H)	1.1%	0.48	CGC	Arg (R)	2.2%	0.37	C
	CUA	Leu (L)	0.3%	0.03	CCA	Pro (P)	0.8%	0.20	CAA	Gln (Q)	1.3%	0.31	CGA	Arg (R)	0.3%	0.05	A
	CUG	Leu (L)	5.2%	0.55	CCG	Pro (P)	2.4%	0.55	CAG	Gln (Q)	2.9%	0.69	CGG	Arg (R)	0.5%	0.08	G
A	AUU	Ile (I)	2.7%	0.47	ACU	Thr (T)	1.2%	0.21	AAU	Asn (N)	1.6%	0.39	AGU	Ser (S)	0.7%	0.13	U
	AUC	Ile (I)	2.7%	0.46	ACC	Thr (T)	1.6%	0.43	AAC	Asn (N)	2.6%	0.61	AGC	Ser (S)	1.5%	0.27	C
	AUA	Ile (I)	0.4%	0.07	ACA	Thr (T)	1.4%	0.30	AAA	Lys (K)	3.8%	0.76	AGA	Arg (R)	0.2%	0.04	A
	AUG	Met (M)	2.6%	1.00	ACG	Thr (T)	1.3%	0.23	AAG	Lys (K)	1.2%	0.24	AGG	Arg (R)	0.2%	0.03	G
G	GUU	Val (V)	2.0%	0.29	GCU	Ala (A)	1.8%	0.19	GAU	Asp (D)	3.3%	0.59	GGU	Gly (G)	2.8%	0.38	U
	GUC	Val (V)	1.4%	0.20	GCC	Ala (A)	2.3%	0.25	GAC	Asp (D)	2.3%	0.41	GGC	Gly (G)	3.0%	0.40	C
	GUA	Val (V)	1.2%	0.17	GCA	Ala (A)	2.1%	0.22	GAA	Glu (E)	4.4%	0.70	GGA	Gly (G)	0.7%	0.09	A
	GUG	Val (V)	2.4%	0.34	GCG	Ala (A)	3.2%	0.34	GAG	Glu (E)	1.9%	0.30	GGG	Gly (G)	0.9%	0.13	G
	U				C				A				G				

Table S3 – Codon usage table for *S. cerevisiae*. %Usage represents overall usage of the codon relative to the total of all codons used for all genes (coding sequences, CDS). Ratio = relative usage based on the frequency that a codon is used for the related amino acid.

	CODON	AMINO ACID	% USAGE	RATIO	CODON	AMINO ACID	% USAGE	RATIO	CODON	AMINO ACID	% USAGE	RATIO	CODON	AMINO ACID	% USAGE	RATIO	
U	UUU	Phe (F)	2.6%	0.59	UCU	Ser (S)	2.3%	0.26	UAU	Tyr (Y)	1.9%	0.56	UGU	Cys (C)	0.8%	0.63	U
	UUC	Phe (F)	1.8%	0.41	UCC	Ser (S)	1.4%	0.16	UAC	Tyr (Y)	1.5%	0.44	UGC	Cys (C)	0.5%	0.37	C
	UUA	Leu (L)	2.6%	0.28	UCA	Ser (S)	1.9%	0.21	UAA	STOP	0.1%	0.47	UGA	STOP	0.1%	0.30	A
	UUG	Leu (L)	2.7%	0.29	UCG	Ser (S)	0.9%	0.10	UAG	STOP	0.05%	0.23	UGG	Trp (W)	1.0%	1.00	G
C	CUU	Leu (L)	1.2%	0.13	CCU	Pro (P)	1.4%	0.31	CAU	His (H)	1.4%	0.64	CGU	Arg (R)	0.6%	0.14	U
	CUC	Leu (L)	0.5%	0.06	CCC	Pro (P)	0.7%	0.15	CAC	His (H)	0.8%	0.36	CGC	Arg (R)	0.3%	0.06	C
	CUA	Leu (L)	1.3%	0.14	CCA	Pro (P)	1.8%	0.42	CAA	Gln (Q)	2.7%	0.69	CGA	Arg (R)	0.3%	0.07	A
	CUG	Leu (L)	1.0%	0.11	CCG	Pro (P)	0.5%	0.12	CAG	Gln (Q)	1.2%	0.31	CGG	Arg (R)	0.2%	0.04	G
A	AUU	Ile (I)	3.0%	0.46	ACU	Thr (T)	2.0%	0.35	AAU	Asn (N)	3.6%	0.59	AGU	Ser (S)	1.4%	0.16	U
	AUC	Ile (I)	1.7%	0.26	ACC	Thr (T)	1.3%	0.22	AAC	Asn (N)	2.5%	0.41	AGC	Ser (S)	1.0%	0.11	C
	AUA	Ile (I)	1.8%	0.27	ACA	Thr (T)	1.8%	0.30	AAA	Lys (K)	4.2%	0.58	AGA	Arg (R)	2.1%	0.48	A
	AUG	Met (M)	2.1%	1.00	ACG	Thr (T)	0.8%	0.14	AAG	Lys (K)	3.1%	0.42	AGG	Arg (R)	0.9%	0.21	G
G	GUU	Val (V)	2.2%	0.39	GCU	Ala (A)	2.1%	0.38	GAU	Asp (D)	3.8%	0.65	GGU	Gly (G)	2.4%	0.47	U
	GUC	Val (V)	1.2%	0.21	GCC	Ala (A)	1.3%	0.22	GAC	Asp (D)	2.0%	0.16	GGC	Gly (G)	1.0%	0.19	C
	GUA	Val (V)	1.2%	0.21	GCA	Ala (A)	1.6%	0.29	GAA	Glu (E)	4.6%	0.70	GGA	Gly (G)	1.1%	0.22	A
	GUG	Val (V)	1.1%	0.19	GCG	Ala (A)	0.6%	0.11	GAG	Glu (E)	1.9%	0.30	GGG	Gly (G)	0.6%	0.12	G
	U				C				A				G				

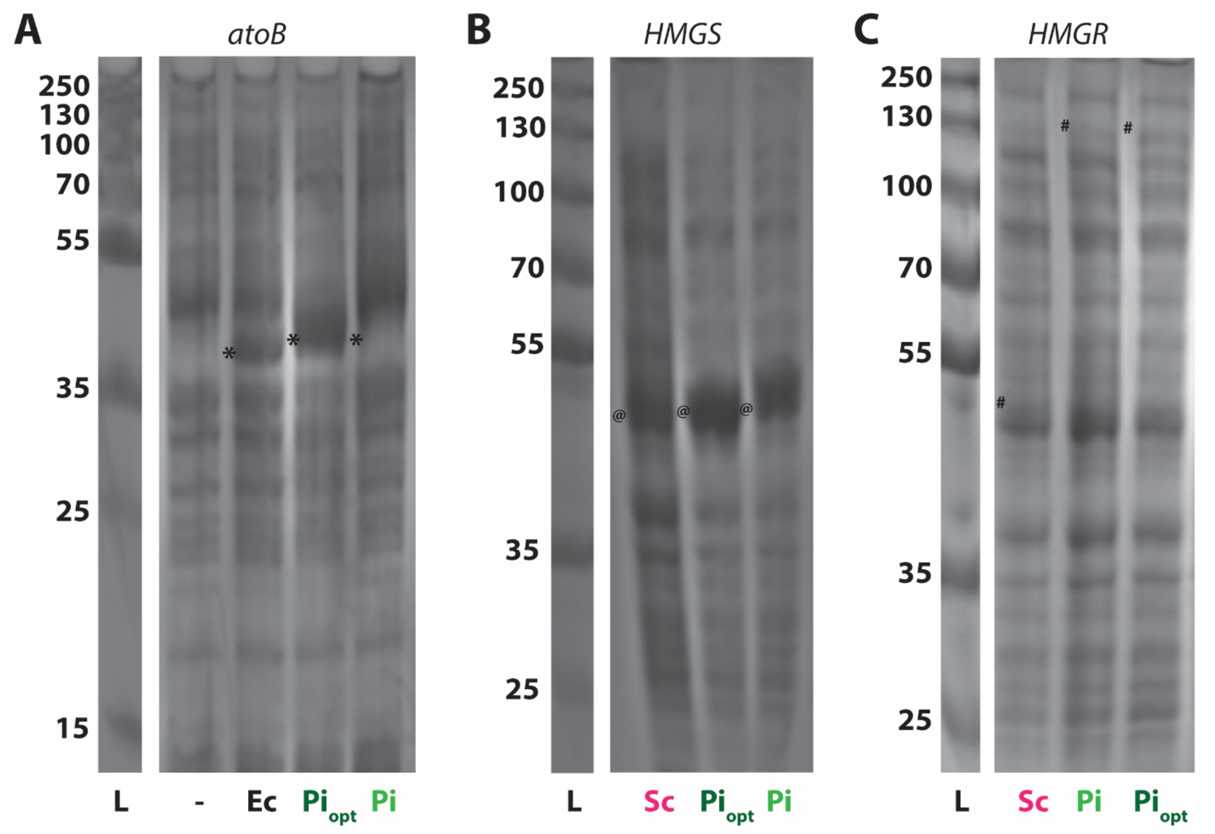

Figure S1 – SDS-PAGE analysis of mevalonate homologs. A) atoB, B) HMGS, and C) HMGR homologs from *E. coli* (Ec), *S. cerevisiae* (Sc), *P. indianae* (Pi) and the *E. coli*-optimized *P. indianae* (Pi_{opt}) gene; * shows the expected sizes/location of the Ec and Pi atoB homologs (40.3 and 43.9 kDa, respectively), @ shows the expected sizes/location of the Sc and Pi HMGS homologs (51.5 and 52.3 kDa, respectively), and # show the expected sizes/location of the Sc and Pi HMGS homologs (53.1 and 112.1 kDa, respectively). L indicates the Page Ruler PLUS ladder and "-" indicates the *E. coli* host control with an uninduced vector. Whole cell lysates of actively growing cultures were analyzed ~ 2 hours after induction and loading was relativized to OD_{600}.

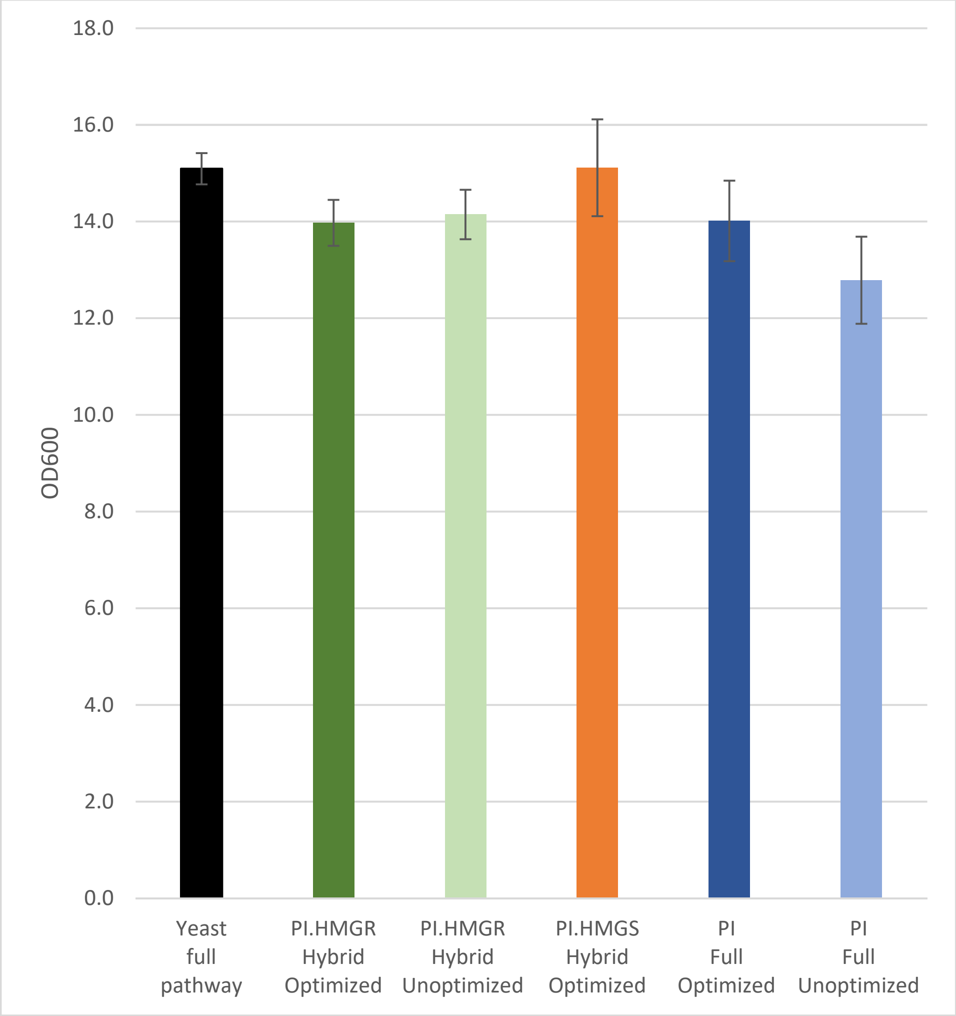

Figure S2: OD of 50 ml cultures containing various mevalonate pathways after 20 hours of growth. Yeast pathway = the *S. cerevisiae* from Martin et al [236]. Full PI pathways = genes form *P. indianae* (Figure 3B) either native or optimized for E. coli. Hybrid pathways use the yeast construct and swap the indicated gene for the original yeast homolog. All pathways evaluated with *atoB*$_{H9}$-*HMGS*$_{C4}$-*HMGR*$_{H9}$ promoter organization. Error bars = standard deviation.

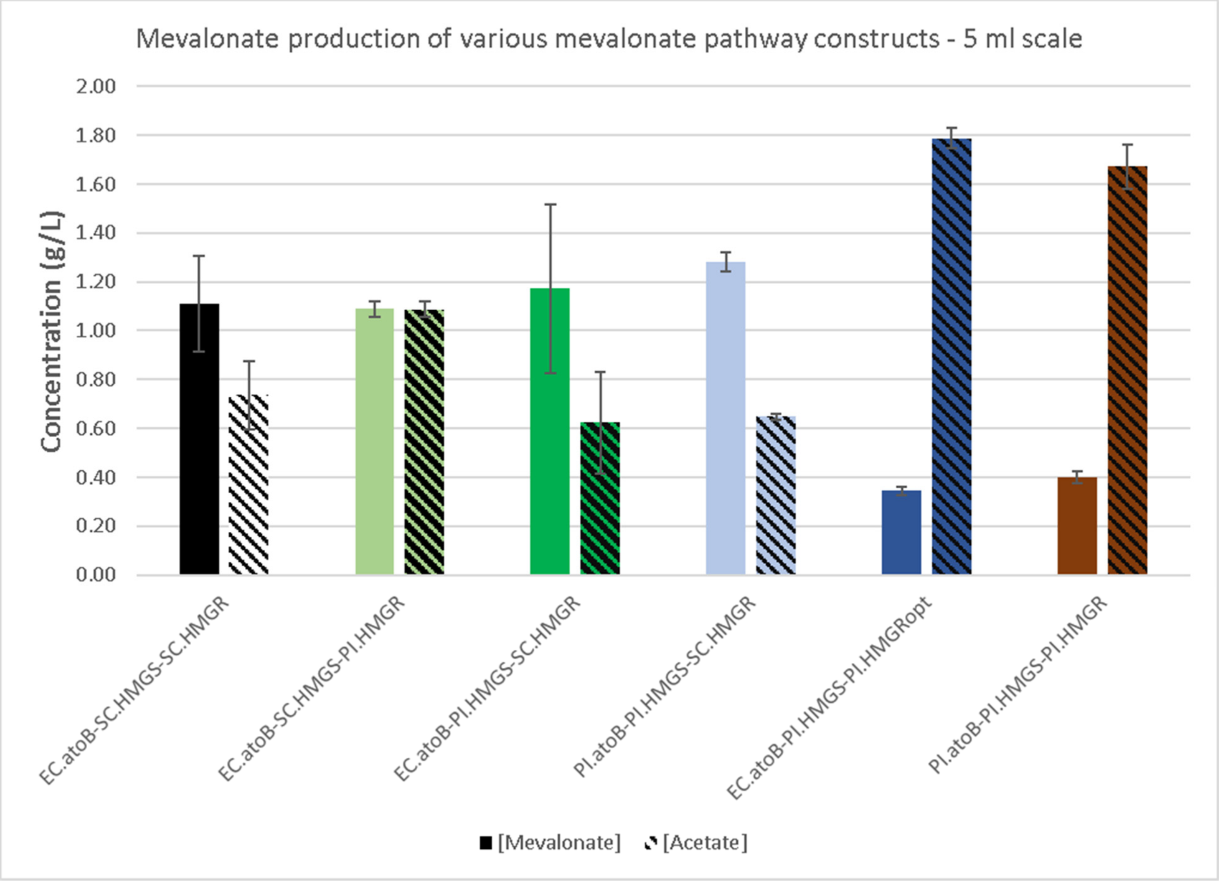

Figure S3: Mevalonate and acetate titers of original and hybrid mevalonate pathways at 5 ml scale. Mevalonate production (solid) and acetate accumulation (striped) from various mevalonate pathway hybrids containing Martin et al (black) or *E. coli*-codon-optimized *P. indianae* genes (various colors, see Figure 6-7) after 20 hrs of culture. All pathways are configured in the high-producing h9-promoter configuration. Errors bars represent standard deviation.

Table S4 – Oligos used in this study

**Name**	**Sequence 5′→3′**	**Purpose**
2.3.1.9_FWD_XhoI	TGGTctcgag**ATG**ACTCTTCAAAAAGATGTTTACATTGTTTCCGC	pETM6-Pi.atoB construction
2.3.1.9_Rev_BcuI	TGGTactagtcccgggyTAAAGAACTTCAATAACGACAGATGAGGC	
2.3.3.10_fwd_XmaI	TGGTctcgagcccggg**ATG**AArGCTAATAAyrTTGGTATTGTT	pETM6-Pi.HMGS construction
2.3.3.10_REV_NotI	TGGTactagtgcggccgcTTATTGrTATmTTTCATArGTTCTrCG	
1.1.1.88_fwd-BglII	TGGTagatctc**ATG**ATGACGACAGTACCwATAGAAG	pETM6-Pi.HMGR construction
1.1.1.88_rev-XhoI	TGGTctcgagTTAAGTATTTTCGAGAACGTCCTTTGAG	
M13F	TGTAAAACGACGGCCAGT	pETM6-Pi.atob, HMGS, & HMGR opt gBLOCK amplification for construction
M13R	CAGGAAACAGCTATGAC	
T7_pro_seq	gtgatgtcggcgatatagg	Promoter Sequencing primer

Start sites bolded, Restrictions sites underlined

Table S5 – *E. coli* strains used in this study

**Name**	**Relevant genotype**	**Source**
DH5a	F– endA1 glnV44 thi-1 recA1 relA1 gyrA96 deoR nupG purB20 φ80dlacZΔM15 Δ(lacZYA-argF)U169, hsdR17(rK–mK+), λ–	ThermoFisher
BL21	F– ompT gal dcm lon hsdSB(rB–mB–) λ(DE3 [lacI lacUV5-T7p07 ind1 sam7 nin5]) [malB+]K-12(λS)	Invitrogen

BL21 CodonPlus RIPL	F– ompT hsdS(rB – mB –) dcm+ Tetr gal λ(DE3) endA Hte [argU proL Camr] [argU ileY leuW Strep/Specr	Agilent Technologies

Table S6 – Gene sequences used in this study.

**Sc.atoB* Silent mutation of NdeI for pETM6 subcloning**
ATGAAAAATTGTGTCATCGTCAGTGCGGTACGTACTGCTATCGGTAGTTTAACGGTT<br>CACTCGCTTCCACCAGCGCCATCGACCTGGGGGCGACAGTAATTAAAGCCGCCATTG<br>AACGTGCAAAAATCGATTCACAACACGTTGATGAAGTGATTATGGGTAACGTGTTAC<br>AAGCCGGGCTGGGGCAAAATCCGGCGCGTCAGGCACTGTTAAAAAGCGGGCTGGCA<br>GAAACGGTGTGCGGATTCACGGTCAATAAAGTATGTGGTTCGGGTCTTAAAAGTGTG<br>GCGCTTGCCGCCCAGGCCATTCAGGCAGGTCAGGCGCAGAGCATTGTGGCGGGGGT<br>ATGGAAAATATGAGTTTAGCCCCCTACTTACTCGATGCAAAAGCACGCTCTGGTTATC<br>GTCTTGGAGACGGACAGGTTTATGACGTAATCCTGCGCGATGGCCTGATGTGCGCCA<br>CCCATGGTTATCACATGGGGATTACCGCCGAAAACGTGGCTAAAGAGTACGGAATTA<br>CCCGTGAAATGCAGGATGAACTGGCGCTACATTCACAGCGTAAAGCGGCAGCCGCA<br>ATTGAGTCCGGTGCTTTTACAGCCGAAATCGTCCCGGTAAATGTTGTCACTCGAAAGA<br>AAACCTTCGTCTTCAGTCAAGACGAATTCCCGAAAGCGAATTCAACGGCTGAAGCGT<br>TAGGTGCATTGCGCCCGGCCTTCGATAAAGCAGGAACAGTCACCGCTGGGAACGCGT<br>CTGGTATTAACGACGGTGCTGCCGCTCTGGTGATTATGGAAGAATCTGCGGCGCTGGC<br>AGCAGGCCTTACCCCCCTGGCTCGCATTAAAAGTTATGCCAGCGGTGGCGTGCCCCC<br>CGCATTGATGGGTATGGGGCCAGTACCTGCCACGCAAAAAGCGTTACAACTGGCGGG<br>GCTGCAACTGGCGGATATTGATCTCATTGAGGCTAATGAAGCATTTGCTGCACAGTTC<br>CTTGCCGTTGGGAAAAACCTGGGCTTTGATTCTGAGAAAGTGAATGTCAACGGCGGG<br>GCCATCGCGCTCGGGCATCCTATCGGTGCCAGTGGTGCTCGTATTCTGGTCACACTAT<br>TACATGCCATGCAGGCACGCGATAAAACGCTGGGGCTGGCAACACTGTGCATTGGCG<br>GCGGTCAGGGAATTGCGATGGTGATTGAACGGTTGAATTAA
**Sc.HMGS**
ATGACTGAACTAAAAAAACAAAAGACCGCTGAACAAAAAACCAGACCTCAAAATGT<br>CGGTATTAAAGGTATCCAAATTTACATCCCAACTCAATGTGTCAACCAATCTGAGCTA<br>GAGAAATTTGATGGCGTTTCTCAAGGTAAATACACAATTGGTCTGGGCCAAACCAAC<br>ATGTCTTTTGTCAATGACAGAGAAGATATCTACTCGATGTCCCTAACTGTTTTGTCTAA<br>GTTGATCAAGAGTTACAACATCGACACCAACAAAATTGGTAGATTAGAAGTCGGTAC<br>TGAAACTCTGATTGACAAGTCCAAGTCTGTCAAGTCTGTCTTGATGCAATTGTTTGGT<br>GAAAACACTGACGTCGAAGGTATTGACACGCTTAATGCCTGTTACGGTGGTACCAAC<br>GCGTTGTTCAACTCTTTGAACTGGATTGAATCTAACGCATGGGATGGTAGAGACGCCA<br>TTGTAGTTTGCGGTGATATTGCCATCTACGATAAGGGTGCCGCAAGACCAACCGGTG<br>GTGCCGGTACTGTTGCTATGTGGATCGGTCCTGATGCTCCAATTGTATTTGACTCTGTA<br>AGAGCTTCTTACATGGAACACGCCTACGATTTTTACAAGCCAGATTTCACCAGCGAAT<br>ATCCTTACGTCGATGGTCATTTTTCATTAACTTGTTACGTCAAGGCTCTTGATCAAGTT<br>TACAAGAGTTATTCCAAGAAGGCTATTTCTAAAGGGTTGGTTAGCGATCCCGCTGGTT<br>CGGATGCTTTGAACGTTTTGAAATATTTCGACTACAACGTTTTCCATGTTCCAACCTGT<br>AAATTGGTCACAAAATCATACGGTAGATTACTATATAACGATTTCAGAGCCAATCCT<br>CAATTGTTCCCAGAAGTTGACGCCGAATTAGCTACTCGCGATTATGACGAATCTTTAA<br>CCGATAAGAACATTGAAAAAACTTTTGTTAATGTTGCTAAGCCATTCCACAAAGAGA<br>GAGTTGCCCAATCTTTGATTGTTCCAACAAACACAGGTAACATGTACACCGCATCTGT

TTATGCCGCCTTTGCATCTCTATTAAACTATGTTGGATCTGACGACTTACAAGGCAAG<br>CGTGTTGGTTTATTTTCTTACGGTTCCGGTTTAGCTGCATCTCTATATTCTTGCAAAATT<br>GTTGGTGACGTCCAACATATTATCAAGGAATTAGATATTACTAACAAATTAGCCAAG<br>AGAATCACCGAAACTCCAAAGGATTACGAAGCTGCCATCGAATTGAGAGAAAATGC<br>CCATTTGAAGAAGAACTTCAAACCTCAAGGTTCCATTGAGCATTTGCAAAGTGGTGTT<br>TACTACTTGACCAACATCGATGACAAATTTAGAAGATCTTACGATGTTAAAAAATAA
**Sc.HMGR* - Silent mutation of NdeI for pETM6 subcloning**
ATGGTTTTAACCAATAAAACAGTCATTTCTGGATCGAAAGTCAAAAGTTTATCATCTG<br>CGCAATCGAGCTCATCAGGACCTTCATCATCTAGTGAGGAAGATGATTCCCGCGATA<br>TTGAAAGCTTGGATAAGAAAATACGTCCTTTAGAAGAATTAGAAGCATTATTAAGTA<br>GTGGAAATACAAAACAATTGAAGAACAAAGAGGTCGCTGCCTTGGTTATTCACGGTA<br>AGTTACCTTTGTACGCTTTGGAGAAAAAATTAGGTGATACTACGAGAGCGGTTGCGGT<br>ACGTAGGAAGGCTCTTTCAATTTTGGCAGAAGCTCCTGTATTAGCATCTGATCGTTTA<br>CCATATAAAAATTATGACTACGACCGCGTATTTGGCGCTTGTTGTGAAAATGTTATAG<br>GTTACATGCCTTTGCCCGTTGGTGTTATAGGCCCCTTGGTTATCGATGGTACATCTTAT<br>CATATACCAATGGCAACTACAGAGGGTTGTTTGGTAGCTTCTGCCATGCGTGGCTGTA<br>AGGCAATCAATGCTGGCGGTGGTGCAACAACTGTTTTAACTAAGGATGGTATGACAA<br>GAGGCCCAGTAGTCCGTTTCCCAACTTTGAAAAGATCTGGTGCCTGTAAGATATGGTT<br>AGACTCAGAAGAGGGACAAAACGCAATTAAAAAAGCTTTTAACTCTACATCAAGAT<br>TTGCACGTCTGCAACATATTCAAACTTGTCTAGCAGGAGATTTACTCTTCATGAGATTT<br>AGAACAACTACTGGTGACGCAATGGGTATGAATATGATTTCTAAAGGTGTCGAATAC<br>TCATTAAAGCAAATGGTAGAAGAGTATGGCTGGGAAGATATGGAGGTTGTCTCCGTT<br>TCTGGTAACTACTGTACCGACAAAAAACCAGCTGCCATCAACTGGATCGAAGGTCGT<br>GGTAAGAGTGTCGTCGCAGAAGCTACTATTCCTGGTGATGTTGTCAGAAAAGTGTTAA<br>AAAGTGATGTTTCCGCATTGGTTGAGTTGAACATTGCTAAGAATTTGGTTGGATCTGC<br>AATGGCTGGGTCTGTTGGTGGATTTAACGCACATGCAGCTAATTTAGTGACAGCTGTT<br>TTCTTGGCATTAGGACAAGATCCTGCACAAAATGTTGAAAGTTCCAACTGTATAACAT<br>TGATGAAAGAAGTGGACGGTGATTTGAGAATTTCCGTATCCATGCCATCCATCGAAG<br>TAGGTACCATCGGTGGTGGTACTGTTCTAGAACCACAAGGTGCCATGTTGGACTTATT<br>AGGTGTAAGAGGCCCGCATGCTACCGCTCCTGGTACCAACGCACGTCAATTAGCAAG<br>AATAGTTGCCTGTGCCGTCTTGGCAGGTGAATTATCCTTATGTGCTGCCCTAGCAGCC<br>GGCCATTTGGTTCAAAGTCACATGACCCACAACAGGAAACCTGCTGAACCAACAAA<br>ACCTAACAATTTGGACGCCACTGATATAAATCGTTTGAAAGATGGGTCCGTCACCTGC<br>ATTAAATCCTAA
**Pi.atoB**
ATGACTCTTCAAAAAGATGTTTACATTGTTTCCGCTGTTCGTACTCCAATTGGTGGTTT<br>AAGTGGTTCTTTAGCTCAATTTAGTGCTACTCAACTTGGTAGCATTGCTATCAAAGGT<br>GCTTTAGAAAAGGCCAATGTTAAGCCAGAAGAAGTTCAAGAAGTATTCTTCGGAAAT<br>GTTTTAACTGCTAACCTTGGTCAAAACCCAGCTCGTCAAGCTGCTTTAGGTGCTGGTA<br>TTCCAAACACTGTTGTTTGTACCACTGTTAACAAGGTTTGTGCTTCCGCTATGAAGGCC<br>ACTATTCTTGGTGCTCAAACTATTATTTAGGTGAAAATGATTTAGTTGTTGTAGGTGG<br>ACAAGAATCTATGACTAACACTCCATACTACATTCCAAAAGCTAGAGCTGGTTGTCG<br>TTACGGTAACCAACAAATTGTTGATGGTATCATTCAAGATGGTCTTTACGATGTTTAC

AACCAATATCAAATGGGTGTTGCTGCTGATGCTACTGCTGCTGAATACAATATCACTA<br>GAAATGATCAAGATGATTTCGCTATTAGAAGTTACAAACTTTCCCAAGAATCCAATG<br>CTAACGGTTATGTTGTCGATGAAATTATTCCAGTTGAAATTCCAGCCACTAAGAGAAC<br>CCCAGCTTCTACTGTTACTACTGATGATGAAATTAACCATCTTAATGAAGCCAAGTTA<br>AGAGCTGTTAGACCAGCTTTCGTCACTGATGGTACTGTTACTGCTCCAAATGCTTCTA<br>CTATTAGTGATGGTGCTGCTGCTCTTGTCTTAGCTTCTAAGGAAAAGGTTGAAGCTTTA<br>GGTTTAAAGCCAATTGCTAAGATTATCGGTTGGGGTGATGCTGCTCATGATCCAGCTC<br>ACTTCACTACTGCTCCATCTTTAGCTATTCCAAAGGCTTTAAAGCATGCTAACAAAAC<br>TCAAGATGAAATTGATTTCTTTGAAATTAATGAAGCTTTCGCTGTTGTTTCCCTTGTTA<br>ACAGTAAGATTTTAAATATTCCAATTGAAAAATTAAATGTCTTTGGTGGTGCTTGTAG<br>TATGGGTCACCCACTTGGTTGTTCCGGTGCTAGAATTATTGTTACTTTAATCAATGTCT<br>TAAAGAAGAAGGGTGGTAAGCTTGGTTGTGCTGCTATCTGTAATGGTGGTGGTGGTGC<br>CTCATCTGTCGTTATTGAAGTTCTTAACCCGGACTAG
**Pi.HMGS**
ATGAAGGCTAATAATGTTGGTATTGTTGCTATGGATATTTACTTCCCAAAACAATACG<br>TTGATCAAGCTCAATTAGAAATTCACGATGGTGCTTCTACTGGTAAATACACTATTGG<br>TTTAGGTCAAACCAGAATGGCTTTCTGTGATGATAGAGAAGATATTAACTCTGTTTGT<br>TTAACTGTTGTTAAGAGTTTAATGGAAAAGTACAACATTGATTACAACCAAGTTGGTG<br>GATTAGAAGTCGGTACTGAAACTATTATTGATAAGTCTAAATCAGTCAAGTCTACTTT<br>AATGCAACTTTTCGCTGAATCTGGAAATACTGATATTGAAGGTATTGATACCACTAAC<br>GCTTGTTATGGTGGTACTAACGCTTTATTCAACACTGTTAACTGGATGGAATCATCTGC<br>TTGGGATGGTCGTTTCGGTTTAGTTGTTGCTGGTGATATTGCTGTTTACGCTTCCGGTA<br>ATGCTAGACCAACCGGTGGTTGTGGTGTTTGTGCTTTATTAATTGGTCCAAACGCTCC<br>AATTGTTATGGAACAAGGTGTCAGAGCTACTCACATGGAAGATGTTTACGATTTCTAC<br>AAGCCAAATCTTGCTTCTGAATTCCCAGAAGTCGATGGTCACTTATCTAACGTTTGTT<br>ACTTAAGATCTGTTGATATTTGTTACAACAGATACATTGAAAAGGTTCAAAAGCGTAC<br>TGGTGAAAAGATTGATATTGAAACCGTTCCATACTTTGTCTTCCACACTCCATACTCTA<br>AGTTAGTCCAAAAATCTTTCGCTAGATTAGCTTTCAACGATTTTATTAAAGACCAAGA<br>AAACCCAAAATACGCTGGTTTAGAAGAACACAAGGGTAAGACTTTAAGTGAAACTT<br>ACTATGATAAACCACTTGAAAAGGCTTTCATGGCTTACACTAAGGAAAAATTCCAAA<br>AGCAAGTTATTCCATCATTATACATTCCAAAGAACTGTGGTAACATGTACTGTGGTTC<br>TGTCTACAGTGCTTTAATTTCTTTAGTTTCTCAAATTCCATCTGAAGAATTATTAAATA<br>AGAGAATTGTTCTCTTCTCTTACGGTTCTGGTTTAGCCGCTTCTATGTACTCATTCAAG<br>GTCATGAATTCTACTGCTGATATTGCTAAGACCTTAAATATTAAGGAAAGATTAGAAA<br>GCAGAAATGAAGTCAAACCAGAAGAATTCGAAAAGATCATGTCTTTAAGAGAAAAG<br>ACTTACCAACTTAAGGATTACACTCCAGTTAGTAAGACTGAAATGTTCCCAGGTACTT<br>ACTATCTTAAGCACGTCGATGAAAAATTCCGCAGAACCTATGAAAGATATCAATAA
**Pi.HMGR**
ATGATGACGACAGTACCTATAGAAGTTAAAACACAAACAGAACAAAAAGTAAATAT<br>TTGGTCTGGATTTTACAAAAAGGCTTTAAAAGAAAGACAAAATCAATTAAAATTAGC<br>ATTTCCAAATCTCTTTCCACCTACTTCACCACTTATATCTAAAGCTTCTTTTCTTCATTC<br>ACCATCCTCAGGATCTGTATCAAATTTAACTGAATCAGCTTCAGATGTTTCAATTCAC<br>CGTAATGAATCTACATCAGATTTAAGCAGTTTAGGTGTTCATGAAGAAAAGTTTCCAA

TTACTTCATTGGATGAACATATTGCTGATAATATGATTGAAAATTGTGTTGGTACTCTT
GGATTGCCAGTTGGTGTTGCTTTAAACTTCAATATTGATAGTAAACCAATTATAGTTCC
AATGGCTATCGAAGAACCATCAGTAGTAGCAGCTGTTTCTGGTGCTGCCAAAACAGT
AGCACAATTCGGTAAAGGAAAAACATTTTATACCAAAACTTCTGAAAGAAATATTAC
TTTTGCCCAAGTGGTTATTTTAGATATTCCGGATAATCGCTTAGATGAAAATGAGAAG
AAGTTAAACGATATGAAAAATGAAATTATTACTTACGCTAATCAATATTGTCAAAAT
ATGTATTCAAGGGGTGGTGGTGTCGTAAATTTAGTAGTTCGTAGAGTTAAGAAGAAC
GAAAGAAAAATTAGACCAAATCAAGTTGTTTTTGATTCTCCATCAAGTGAATGGTTAG
TATGTCATTTTCATATTGATGTTTGTGATGCTATGGGTGCAAACTGTGCTAGTACTGTT
GCAGAAGGTGTCGCACCATTCCTTGCTGATTTAACAAATGGTCGTATTGGATTAAGAA
TTGTATCCAATTTATGTACTGAAAGAATTGTTACTGCTTCTTTCAAAATTCCAGTTGAA
AAAATGAAATACAAAAAATTCACTGGTGAACAAGTTTCAAGAGGTATTATAGAAGC
ATATGAATTCGCTGAAGATGATGTTTACAGAGCTACTACCCATAATAAGGGTATTTTA
AATGGTATCGATGCTGTTGCCTTAGCAACTGGTCAAGACTGGAGAGCTATTGAAGCT
GCTGCACATGCTTATGCTGCTTCTGGTTCTAGTGTAACTGTTAATGAACCAGTTAAAG
GTCATTATAAATCTTTAACTAGTTATTGGATGGAAGAAGTTGAAGAAGAAGTAGATA
ATCAAAAAACTAAACAACGTTACTTCTGTGGTGAAATTAAATTACCAATTGCTGTTGG
TACTAAGGGTGGTGTATTAAAAACAAATCCAGTTTATCATTACACTTTAGGTCTTATG
GGTCATCCAGATTCTAAGGCTTTGTCAGCTATTTTTGCTTGTGTTGGTCTTGCTCAAAA
CTTTGCTGCTGTTAGAGCTTTAACAACTGAAGGTATTCAAAGAGGTCACATGTCTCTT
CACGCTCGTAATATTGCTATTGCTGCAGGTGCTCCATCTCATGCTATTGCTGAAGTTAC
TGATTATATGGTAGCATGCAATAGAATTAATTTAAATGCTGCCAAGGAATACTTATTA
GCTCACGAATTACATAGTACTTTGAGAAAGCATTTAGAAGGTTCTGATTCCCAATCAT
CAAAACCACCAAGTATGTTCTATTTTGAAGAATATGTTCCAGAAGGTGAAGATAAAA
ACAATCGTATCACCTTAAACATTGCCTTCCAAACATTAACTGATCAACCAACTAATAT
TGAATATCCAACTGGATCAAATGATGATCCAATATCTCAACTTTTATTTGGTAATAAA
GATTACACATGGATTTCATCAATGCTTAATGTATTAGATAAATTCCAATTTAGTACAG
CTTCTCAAGGACGTGCTAACTTTGTTTTAGCAAAAAAATTAAAACTCTTATCAATGCT
TATCAATATAATTACTACTAGATTAATGACTTACTATCCAAAACAAACTACTCGTTTT
ATTGAAAGAATCTTTAGACATTCAAAGAGACCAAAGTCAAGTACTTCATCATCCTTA
AAAACTAAAACTCAACAACCACCATCAATTGAAAAATATCTTTTAAGTATTCAAATT
TCTGCTAATCAACAACTTCAAAACCAAAATATTCACTTACCTGGTCCAATTTCTGCTC
ATTCTGTTCCAAGTAGCAATGGTTTATCTTGGTCTGTTCCAGATACTGGTATTTTCCGT
AAGAATATTTTAGATATTGCAGATGATGTTAAAAATTTAAGTGATCCAACTTTAATTC
AAGTAGGTTTTCCATTATTATTGGCTTTATGGCAAGTTTTTGAATTAAGAGTAGTTCAA
TGGGTTGGTCATTCTTCACTTTCATCATCACTTCTTGAAGAACAAAGAAAAGTTATAA
GTTCAATTGTTTCTTCACCAATTCCACCATACAAACCAAGTGAAGTCCGTCCACCAGT
TATAATTAATAATACTGCAAAGGTTCAAGAACCACCTTATCTTAGTTCCTTCAACCGT
TTCATTCCAATTTATTCAAAGAGATTCCAAGTTACAATGATTTTACTTTGTAATGCTAT
ATCTTTTGATCCAACTCTTATTAACTCCCGTAGAATTAAATTCTTATTAACATTAGGTA
GTTACTTAGAATGGGAATTAGCTAAGGATCATGATCTTGGTCGTCTAGGACGTGATCT
TATGCTTGTTCAAAACCAAAATCAAAAAATTAGTCACGATGGTAGTATTGGTAATGGT
ATCACAAACTCATTCATCATTTGGAAGGAAATGCGTGAAAAAACTAATCCAAAACAA

AATATGTTCCACTTTGAAGATGATGATGATACTGATGATTCTGCTAGTACCCCATCTA<br>TATCTAATATAGTTGAATCTGTAGATAATGAACCATACTATCAACGTTATAATGATGA<br>AATTAAACAATATTTATTAGAAACTGATCATTCACAAATAAAATCACAATTATTTAAT<br>ACTGAATCATGTCTTTTATTCTCAAAGGACGTTCTCGAAAATACTTAA
**Pi.atoB-opt**
ATGACTCTCCAGAAAGATGTGTATATTGTTTCCGCGGTGCGTACCCCAATCGGCGGAT<br>TAAGCGGTTCTTTGGCCCAATTTAGTGCAACACAGCTTGGTAGCATTGCGATCAAAGG<br>GGCTCTGGAAAAGGCCAATGTTAAGCCGGAGGAAGTTCAGGAAGTATTTTTCGGCAA<br>TGTGCTGACGGCGAACCTGGGTCAAAACCCGGCGCGTCAGGCAGCCCTGGGCGCGG<br>GTATTCCAAACACTGTGGTTTGTACCACCGTGAACAAAGTTTGCGCTTCCGCAATGAA<br>AGCCACAATCCTGGGCGCGCAGACGATTATCTTGGGTGAGAACGATCTGGTGGTAGT<br>AGGGGGACAAGAATCTATGACTAACACACCGTACTATATCCCGAAAGCCCGCGCGG<br>GCTGCCGTTACGGTAACCAGCAGATTGTGGACGGCATCATTCAGGATGGCCTTTACG<br>ATGTGTACAACCAGTATCAAATGGGGGTTGCAGCTGACGCGACCGCCGCAGAATACA<br>ATATCACGCGTAATGACCAGGATGATTTCGCGATTCGCAGCTATAAACTGTCCCAGG<br>AATCCAATGCTAACGGTTATGTGGTCGATGAAATCATTCCAGTAGAAATCCCGGCCA<br>CTAAACGCACCCCAGCGTCTACAGTGACGACCGATGACGAAATTAACCATCTTAACG<br>AAGCCAAATTACGTGCAGTTCGCCCGGCCTTTGTCACTGATGGCACAGTGACGGCGC<br>CGAATGCTTCTACCATCAGCGATGGTGCAGCGGCCCTGGTCCTGGCGTCTAAAGAAA<br>AAGTAGAGGCATTGGGCCTGAAGCCGATTGCTAAAATCATCGGGTGGGGCGATGCGG<br>CCCATGACCCGGCACACTTCACTACCGCGCCATCTCTGGCTATCCCAAAGGCGCTGA<br>AACATGCAAACAAAACGCAAGATGAGATTGACTTCTTTGAAATCAATGAGGCCTTCG<br>CGGTGGTTTCCCTGGTGAACAGCAAAATTTTAAATATCCCGATTGAAAAATTGAATGT<br>CTTTGGCGGTGCTTGCAGCATGGGCCACCCGCTGGGGTGTTCCGGTGCACGCATCATT<br>GTTACACTGATCAACGTCTTGAAAAAAAAGGGCGGTAAACTGGGCTGCGCGGCCATC<br>TGTAATGGTGGCGGCGGTGCCTCATCTGTCGTGATTGAAGTACTTAACCCGGATTAG
**Pi.HMGS-opt**
ATGAGCGGTATGAAAGCCGAGAATGTTGGCATCGTGGCGATGGACATCTACTTCCCG<br>AAGCAGTACGTGGATCAAGCCCAACTGGAAATCCATGATGGCGCCAGCACGGGCAA<br>ATATACCATTGGTCTGGGCCAGACCCGCATGGCGTTCTGTGATGACCGTGAGGACAT<br>CAACAGTGTGTGTCTGACGGTGGTGAAGAGTCTGATGGAAAAGTACAACATCGACTA<br>TAACCAAGTTGGCCGTCTGGAAGTGGGCACCGAGACGATCATCGACAAGAGCAAGA<br>GCGTGAAGAGCACGCTGATGCAGCTGTTTGCGGAAAGCGGTAACACCGACATCGAA<br>GGCATCGACACCACCAACGCGTGCTACGGTGGCACGAACGCGCTGTTCAACACGGTG<br>AACTGGATGGAAAGCAGTGCGTGGGATGGTCGCTTCGGTCTGGTGGTTGCGGGCGAT<br>ATCGCCGTGTATGCCAGCGGTAATGCGCGCCCAACGGGTGGTTGCGGCGTTTGTGCG<br>CTGCTGATCGGCCCGAATGCGCCGATCGTTATGGAACAAGGCGTGCGCGCGACCCAC<br>ATGGAGGATGTTTACGACTTCTACAAACCGAATCTGGCCAGCGAGTTTCCAGAAGTG<br>GACGGCCATCTGAGCAATGTGTGCTATCTGCGCAGCGTGGACATCTGCTACAATCGCT<br>ACATCGAGAAAGTGCAGAAGCGTACCGGCGAGAAGATCGATATCGAGACGGTGCCG<br>TACTTCGTGTTTCATACGCCGTACAGCAAGCTGGTGCAGAAAAGCTTTGCCCGCCTCG<br>CCTTTAACGACTTCATCAAGGATCAAGAAAACCCAAAATACGCGGGTCTGGAAGAA

CACAAAGGCAAGACCCTCAGCGAGACCTACTACGATAAGCCGCTGGAGAAAGCCTT<br>CATGGCGTACACCAAGGAGAAGTTCCAGAAACAAGTTATTCCGAGTCTCTACATCCC<br>GAAGAATTGCGGCAACATGTACTGCGGCAGCGTTTACAGCGCCCTCATCAGTCTGGT<br>GAGCCAGATCCCGAGTGAAGAGCTGCTCAACAAGCGCATCGTGCTCTTCAGCTATGG<br>TAGCGGTCTGGCGGCCAGCATGTACAGCTTCAAGGTGATGAACAGCACCGCCGACAT<br>CGCGAAGACGCTCAACATCAAGGAACGTCTGGAGAGTCGCAACGAAGTTAAGCCGG<br>AGGAGTTCGAGAAGATCATGAGTCTCCGCGAGAAGACCTACCAGCTCAAAGACTAC<br>ACGCCGGTTAGCAAGACCGAGATGTTTCCGGGCACCTACTATCTGAAGCACGTTGAT<br>GAGAAGTTCCGCCGCACCTACGAACGTTACCAGTAA
**Pi.HMGR-opt**
ATGATGACGACAGTCCCGATCGAAGTTAAGAGCCAGACGGAACAAAAAGTAAACAT<br>CTGGTCTGGTTTTTACAAAAAAGCGCTGAAGGAACGCCAGAATCAGTTGAAACTGGC<br>CTTTCCGAACTTATTCCCGCCGATTTCACCACTCATCAGCAAAACCTCCTTCCTTAATT<br>CGCCGAGCCTGGGCTCACTGTCTAACCTGACGGAGAGCGCGTCCGATGTGTCAATTA<br>ACCGTAATGAATCGACTTCCGATTTATCTTCCTTGGGTGTCCATGAAGAAAAATTTCC<br>GATTACAAGCCTGGACGAGCACATCGCGGATAACATGATTGAAAACTGTGTAGGCAC<br>GCTCGGGCTGCCTGTTGGTGTCGCACTGAATTTTAACATCGATAACAAATCAATTATT<br>GTGCCGATGGCCATCGAAGAACCATCGGTGGTTGCAGCTGTAAGCGGCGCAGCCAA<br>AACCGTGTCTCAATTCGGTAAAGGAAAAACCTTTTTCACCAAAACCTCCGAGCGTAA<br>TATTACTTTTGCACAGGTCGTGATCCTGGACATTCCAGATGATAAATTGGACGAAAAC<br>GAAAAGAAGCTGAATGATATGAAAAACGAAATCATTATCTTTGCCAATCAGTATTGC<br>CAAAACATGTACTCTCGCGGCGGTGGCGTTGTCAATATGGTAGTGCGTCGGATTAAA<br>AAGAACGAGCGCAAAGTACGCCCGAACCAGGTAGTTTTTGATAGCCAGTCCAGTGA<br>ATGGTTAGTGTGTCATTTCCACATCGACGTTTGCGATGCAATGGGGGCGAATTGCGCT<br>TCTACAGTGGCAGAAGGTGTTGCACCGTTCCTTGCCGATCTGACAAACGGCCGTATTG<br>GACTGCGCATCGTGTCTAATCTGTGTACGGAAAGAATTGTCACCGCGTCGTTTAAGAT<br>TCCTGTTGAGAAAATGAAATACAAGAAATTTACAGGTGAACAAGTAAGCCGCGGCA<br>TCATTGAGGCTTATGAATTTGCGGAAGACGATGTGTACCGCGCAACCACTCATAACA<br>AAGGTATCCTGAACGGCATTGATGCCGTTGCTCTGGCAACCGGTCAGGACTGGCGCG<br>CCATCGAGGCGGCGGCACACGCATACGCTGCGAGCGGCTCCAGTGAGACTGTGAAT<br>GAACCGGTAAAGGGTCATTATAAATCATTAACCAGTTACTGGATCGAGGAGATCGAA<br>GAAGAGATCAACAACGAAAAGGTTAAACAGCGTTACTTCTGCGGGGAAATTAAAAT<br>GCCCATTGCGGTGGGTACAAAGGGCGGTGTCCTGAAAACCAACCCTGTTTATCACTA<br>CACTCTGGGTCTAATGGGCAATCCAGATTCTAAAGCGCTGTCCGCTATTTTCGCCTGT<br>GTGGGTTTGGCTCAAAACTTTGCCGCAGTACGTGCGCTGACGACAGAAGGCATTCAG<br>CGCGGTCACATGAGCCTCCATGCTCGAAATATCGCCATTGCGGCGGGTGCTCCGAGC<br>CACGCAATTGCGGAAGTCACTGATTATATGGTTGCaTGCAACCGCATCAACCTGAATG<br>CCGCGAAAGAGTACTTGTTAGCGCATGAACTGCACAGTACCCTGCGTAAACATCTGG<br>ACGGCACCGATCCTCAGTCATCCAAACCGCCGAGTATGTTCTATTTCGAAGAATACAT<br>TCCAGAGGGCGAAGATAAAAACAACCGTATCACCCTGAATATTGCCTTCCAAACCCT<br>GACTGACCAGCCAACAAACATCGAATATCCGACCGGGagcAATGACGATCCGGTTTCC<br>CAGCTTTTATTTGGCAACAAGGACTACACTTGGATAACGTCGATGTTGAACGTTCTGG<br>ATAAATTCCAGTTTAATACAGCGTCTCAAGGTCGTGCCAACCTCGTGCTGGCGAAAA

AACTGAAACTGCTGtcaATGCTTATTAACATAATTACCACTCGGTTAATGACGTATTAT
CCAAAACAGACTGTTCGTTTCATCGAACGCATCTTTCGTCACAGCAAACGCCCGAAG
TCAACTTCTACATCCTCTGTAAAAACCAAAAATCAGCAGCCGCCGAGCATTGAGAAA
TACCTTCTGAGTATTCAAATCTTTGCCAACCAGCATTTGCAGAATCAGAACATTCACT
TGCCAGGCCCTATTAGCGCTCATTCTGTGCCGAGCAGTAGTGGTCTAAGCTGGTCTGT
CCCGGATACCGGGATCTTCCGTAAGAATATCCTGGACATTGCGGATGATGTTAAAAA
CTTAAGTGACCCGACGCTGATCCAAGTGGGCTTCCCACTGCTGCTGGCTCTGTGGCAG
GTTTTTGAACTGCGAGTCGTGCAGTGGGTTGGCCACTCAAGCCTTTCTTCCAGCCTCCT
TGAAGAACAACGCAAAGTGATTAGTTCGATTGTAAGCTCACCTATCCCGCCGTATAA
ACCGAGCGAGGTTCGCCCGCCGATTATCATCAATGGCAACTCTAAAGTGCAGGAACA
ACCATATTTGAACAGCTTTAACCGTTTCGTTCCCATCTATTCCAAACGTTTCCAGGTGA
CGATGATACTGCTCTGTAATGCGATCTCCTTTGATCCTACGCTTATTAACTCTCGTCGG
ATTAAATTCCTGCTGACACTGGGCAGTTACCTGGAATGGGAATTAGCTAAAGATCAT
GACCTCGGTCGCCTGGGACGTGACCTCATGTTGATCCAGAATTACAACAAAAAGGTT
AGCCATGATGGCTCTATTGGTAATGGGATCACAAACAGCTTTATTATCTGGAAAGAG
ATGCGTGAAAAAACGAACCCGAAACAGAATATGTTCCACTTCGAAGACGATGAAGA
TACAGACGATTCAATTAGCGTGAGCACACCTTCCATTTCTAACATCGTCGAAAGCGTT
GACAATGAGTCGTACTATCAACGTTATAATGACGAAATCAAACAGTATCTGCTGGAA
ACTGATCATTCTGAGATTAAATCGCAGCTGTTTAACACGGAGAGCTGCCTTCTGTTCT
CCAAGGACGTGCTCGAAAACACTATTGAAGAGTACCAGAAGTATTACAATGTGAAA
AACCTGCTGAACAACCTGAATACGAACTAA

Table S7 – Plasmids used in this study.

Name	Relevant Phenotype	Plasmid origin of replication	Source
pETM6-T7-mCherry	AmpR, lacO/I, P_{T7} mCherry	ColE1 (pBR322)	Xu et al., 2012
pETM6-H9-mCherry	AmpR, lacO/I, P_{H9} mCherry	ColE1 (pBR322)	Jones et al., 2015b
pETM6-C4-mCherry	AmpR, lacO/I, P_{CA} mCherry	ColE1 (pBR322)	Jones et al., 2015b
pMevT	AmpR, lacO/I, P_{lac}-Ec.atoB-Sc.HMGS-Sc.HMGR	p15a	Martin et al, 2003
pETM6-T7-Ec.atoB	AmpR, lacO/I, P_{T7} Ec.atoB	ColE1 (pBR322)	This Study
pETM6-H9-Ec.atoB	AmpR, lacO/I, P_{H9} Ec.atoB	ColE1 (pBR322)	This Study
pETM6-T7-Sc.HMGS	AmpR, lacO/I, P_{T7} Sc.HMGS	ColE1 (pBR322)	This Study
pETM6-C4-Sc.HMGS	AmpR, lacO/I, P_{C4} Sc.HMGS	ColE1 (pBR322)	This Study
pETM6-T7-Sc.HMGR	AmpR, lacO/I, P_{T7} Sc.HMGR	ColE1 (pBR322)	This Study
pETM6-H9-Sc.HMGR	AmpR, lacO/I, P_{H9} Sc.HMGR	ColE1 (pBR322)	This Study
pETM6-H9-Ec.atoB_C4-Sc.HMGS	AmpR, lacO/I, P_{H9} Ec.atoB, P_{C4} Sc.HMGS	ColE1 (pBR322)	This Study
pETM6-H9-Ec.atoB_C4-Sc.HMGS_H9-Sc.HMGR	AmpR, lacO/I, P_{H9} Ec.atoB, P_{C4} Sc.HMGS, P_{H9} Sc.HMGR	ColE1 (pBR322)	This Study
pETM6-H9-Ec.atoB_C4-Sc.HMGS_H9-Pi.HMGR	AmpR, lacO/I, P_{H9} Ec.atoB, P_{C4} Sc.HMGS, P_{H9} Pi.HMGR	ColE1 (pBR322)	This Study
pETM6-T7-Pi.atoB	AmpR, lacO/I, P_{T7} Pi.atoB	ColE1 (pBR322)	This Study
pETM6-H9-Pi.atoB	AmpR, lacO/I, P_{H9} Pi.atoB	ColE1 (pBR322)	This Study
pETM6-T7-Pi.HMGS	AmpR, lacO/I, P_{T7} Pi.HMGS	ColE1 (pBR322)	This Study
pETM6-C4-Pi.HMGS	AmpR, lacO/I, P_{C4} Pi.HMGS	ColE1 (pBR322)	This Study

pETM6-T7-Pi.HMGR	AmpR, lacO/I, P_{T7} Pi.HMGR	ColE1 (pBR322)	This Study
pETM6-H9-Pi.HMGR	AmpR, lacO/I, P_{H9} Pi.HMGR	ColE1 (pBR322)	This Study
pETM6-T7-9-AGF_T7-10-AGF	AmpR, lacO/I, P_{T7} Pi.atoB, P_{T7} Pi.HMGS	ColE1 (pBR322)	This Study
pETM6-H9-9-AGF_T7-10-AGF	AmpR, lacO/I, P_{H9} Pi.atoB, P_{T7} Pi.HMGS	ColE1 (pBR322)	This Study
pETM6-T7-9-AGF_T7-10-AGF_T7-88-AGF	AmpR, lacO/I, P_{T7} Pi.atoB, P_{T7} Pi.HMGS, P_{T7} Pi.HMGR	ColE1 (pBR322)	This Study
pETM6-H9-9-AGF_T7-10-AGF_H9-88-AGF	AmpR, lacO/I, P_{H9} Pi.atoB, P_{T7} Pi.HMGS, P_{H9} Pi.HMGR	ColE1 (pBR322)	This Study
pETM6-T7-Pi.atoB-opt	AmpR, lacO/I, P_{T7} Pi.atoB-opt	ColE1 (pBR322)	This Study
pETM6-H9-Pi.atoB-opt	AmpR, lacO/I, P_{H9} Pi.atoB-opt	ColE1 (pBR322)	This Study
pETM6-T7-Pi.HMGS-opt	AmpR, lacO/I, P_{T7} Pi.HMGS-opt	ColE1 (pBR322)	This Study
pETM6-C4-Pi.HMGS-opt	AmpR, lacO/I, P_{C4} Pi.HMGS-opt	ColE1 (pBR322)	This Study
pETM6-T7-Pi.HMGR-opt	AmpR, lacO/I, P_{T7} Pi.HMGR-opt	ColE1 (pBR322)	This Study
pETM6-H9-Pi.HMGR-opt	AmpR, lacO/I, P_{H9} Pi.HMGR-opt	ColE1 (pBR322)	This Study
pETM6-H9-Ec.atoB_C4-Sc.HMGS_H9-Pi.HMGR-opt	AmpR, lacO/I, P_{H9} Ec.atoB, P_{C4} Sc.HMGS, P_{H9} Pi.HMGR-opt	ColE1 (pBR322)	This Study
pETM6-H9-Pi.atoB-opt_C4-Pi.HMGS-opt_H9-Sc.HMGR	AmpR, lacO/I, P_{H9} Pi.atoB, P_{C4} Pi.HMGS, P_{H9} Sc.HMGR-opt	ColE1 (pBR322)	This Study
pETM6-H9-Pi.atoB-opt_C4-Pi.HMGS-opt_H9-Pi.HMGR-opt	AmpR, lacO/I, P_{H9} Pi.atoB, P_{C4} Pi.HMGS, P_{H9} Pi.HMGR-opt	ColE1 (pBR322)	This Study
pETM6-H9-Ec.atoB_C4-Pi.HMGS-opt_H9-Sc.HMGR	AmpR, lacO/I, P_{H9} Ec.atoB, P_{C4} Pi.HMGS-opt, P_{H9} Sc.HMGR	ColE1 (pBR322)	This Study
pETM6-H9-Ec.atoB_C4-Pi.HMGS-opt_H9-Pi.HMGR-opt	AmpR, lacO/I, P_{H9} Ec.atoB, P_{C4} Pi.HMGS-opt, P_{H9} Pi.HMGR-opt	ColE1 (pBR322)	This Study
pETM6-H9-Pi.atoB-opt_C4-Sc.HMGS_H9-Sc.HMGR	AmpR, lacO/I, P_{H9} Pi.atoB-opt, P_{C4} Sc.HMGS, P_{H9} Sc.HMGR	ColE1 (pBR322)	This Study
pETM6-H9-Pi.atoB-opt_C4-Sc.HMGS_H9-Pi.HMGR-opt	AmpR, lacO/I, P_{H9} Pi.atoB-opt, P_{C4} Sc.HMGS, P_{H9} Pi.HMGR-opt	ColE1 (pBR322)	This Study
